# Supplementary material for: Regression assumptions in clinical psychology research practice—a systematic review of common misconceptions
Source: PeerJ. 2017 May 16;5:e3323. doi: 10.7717/peerj.3323 (PMC5436580; doi:10.7717/peerj.3323)
Supplement: Supplemental Information 1 — This supplementary material outlines the individual classification for each of the papers analyzed. [file peerj-05-3323-s001.pdf]

Supplementary Material to ‘Regression Assumptions in Clinical Psychology Research Practice A systematic review of common misconceptions’

Anja F. Ernst & Casper J. Albers

Abstract

In our paper ‘Regression Assumptions in Clinical Psychology Research Practice A systematic review of common misconceptions’ we analyse nearly 1,000 articles, from 12 different journals in clinical psychology. Summaries of our results are provided in the tables our our paper. This supplementary material outlines the individual classification for each of the papers analysed.

Part I

Q1 top 3 Clinical Journals: Annual Review of Clinical Psychology, Clinical Psychology Review, Journal of Consulting and Clinical Psychology

1 No Model of Interest

Annual Review of Clinical Psychology

1. Interventions for Tobacco Smoking; Tanya R. Schlam, Timothy B. Baker
2. Neurotoxic Effects of Alcohol in Adolescence; Joanna Jacobus, Susan F. Tapert
3. Socioeconomic Status and Health: Mediating and Moderating Factors
4. The Manufacture of Recovery; Joel Tupper Braslow
5. How Can We Use Our Knowledge of Alcohol-Tobacco Interactions to Reduce Alcohol Use? Sherry A. McKee and Andrea H. Weinberger
6. Revisiting the Relationship Between Autism and Schizophrenia: Toward an Integrated Neurobiology Nina de Lacy and Bryan H. King

7. The Relationship Between Personality Disorders and Axis I Psychopathology: Deconstructing Comorbidity Paul S. Links, Rahel Eynan
8. Psychosocial Treatments for Schizophrenia Kim T. Mueser, Frances Deavers, David L. Penn, Jeffrey E. Cassisi
9. Cognitive Interventions Targeting Brain Plasticity in the Prodromal and Early Phases of Schizophrenia; Melissa Fisher, Rachel Loewy, Kate Hardy, Danielle Schlosser, Sophia Vinogradov
10. Emotion Deficits in People with Schizophrenia; Ann M. Kring, Ori Elis
11. Postpartum Depression: Current Status and Future Directions; Michael W. OHara, Jennifer E. McCabe
12. Depression and Cardiovascular Disorders; Mary A. Whooleya, Jonathan M. Wong
13. Worry and Generalized Anxiety Disorder: A Review and Theoretical Synthesis of Evidence on Nature, Etiology, Mechanisms, and Treatment Michelle G. Newman, Sandra J. Llera, Thane M. Erickson, Amy Przeworski, Louis G. Castonguay
14. Social Anxiety and Social Anxiety Disorder; Amanda S. Morrison and Richard G. Heimberg
15. Fear Extinction and Relapse: State of the Art; Bram Vervliet, Michelle G. Craske, Dirk Hermans
16. Endophenotypes in Psychopathology Research: Where Do We Stand? Gregory A. Miller and Brigitte Rockstroh
17. Ambulatory Assessment Timothy J. Trull, Ulrich Ebner-Priemer
18. Principles Underlying the Use of Multiple Informants Reports; Andres De Los Reyes, Sarah A. Thomas, Kimberly L. Goodman, Shannon M.A. Kundey
19. Network Analysis: An Integrative Approach to the Structure of Psychopathology Denny Borsboom and Angelique O.J. Cramer
20. Integrative Data Analysis in Clinical Psychology Research Andrea M. Hussong, Patrick J. Curran, and Daniel J. Bauer
21. Dependent personality disorder: A critical review Krystle L. Disney
22. Bringing the Laboratory and Clinic to the Community: Mobile Technologies for Health Promotion and Disease Prevention; Robert M. Kaplan,Arthur A. Stone
23. Detecting Consciousness: A Unique Role for Neuroimaging; Adrian M. Owen
24. Shifting Gears: Seeking New Approaches for Mind/Brain Mechanisms; Michael S. Gazzaniga
25. The Endocannabinoid System and the Brain; Raphael Mechoulam and Linda A. Parker)
26. Evidence-Based Psychological Treatments: An Update and a Way Forward David H. Barlow, Jacqueline R. Bullis, Jonathan S. Comer, and Amantia A. Ametaj
27. Psychosocial treatments for negative symptoms in schizophrenia: Current practices and future directions Ori Elis, Janelle M. Caponigro, Ann M. Kring

28. Acceptance and Commitment Therapy in the treatment of anxiety: A systematic review; Jessica Swain, Karen Hancock,Cassandra Hainsworth, JennyBowman
29. School Bullying: Development and Some Important Challenges
30. Quitting Drugs: Quantitative and Qualitative Features; Gene M. Heyman
31. Stability and Change in Personality Disorders Leslie C. Morey, Christopher J. Hopwood
32. The Genetics of Eating Disorders; Sara E. Trace, Jessica H. Baker, Eva Penas-Lled, Cynthia M. Bulik
33. Dissociative Disorders in DSM-5; David Spiegel, Roberto Lewis-Fernandez, Ruth Lanius, Eric Vermetten,Daphne Simeon, Matthew Friedman

Clinical Psychology Review

1. Interventions for caregivers of children who disclose sexual abuse: A review; Annik van Toledo, Fred Seymour
2. Treatment development: Can we find a better way? Steven C. Hayes, Douglas M. Long, Michael E. Levin, William C. Follette
3. A systematic review on the effectiveness of sex offender risk assessment tools in predicting sexual recidivism of adult male sex offenders; Ruth J. Tully, Shihning Chou, Kevin D. Browne
4. Pretrauma risk factors for posttraumatic stress disorder: A systematic review of the literature; Julia A. DiGangi, Daisy Gomez, Leslie Mendoza, Leonard A. Jason, Christopher B. Keys, Karestan C. Koenen
5. What should we expect from psychotherapy? Marvin R. Goldfried
6. A systematic review of randomised controlled trials of interventions reporting outcomes for relatives of people with psychosis Fiona Lobban; Adam Postlethwaite, David Glentworth, Vanessa Pinfold, Laura Wainwright, Graham Dunn, Anna Clancy, Gillian Haddock
7. Cognitive-behavioral therapy versus other therapies: Redux; Timothy P. Baardseth, Simon B. Goldberg, Brian T. Pace, Andrew P. Wislocki, Nick D. Frost, Jamila R. Siddiqui, Abigail M. Lindemann, D. Martin Kivlighan III, Kevin M. Laska, Aaron C. Del Re, Takuya Minami, Bruce E. Wampold
8. A Cognitive Attachment Model of prolonged grief: Integrating attachments, memory, and identity; Fiona Maccallum, Richard A. Bryant
9. Neuroticism and common mental disorders: Meaning and utility of a complex relationship; Johan Ormel, Bertus F. Jeronimus, Roman Kotov, Harritte Riese, Elisabeth H. Bos, Benjamin Hankin, Judith G.M. Rosmalen, Albertine J. Oldehinkel
10. Neuroticism and common mental disorders: Meaning and utility of a complex relationship Johan Ormel, Bertus F. Jeronimus, Roman Kotov, Harritte Riese, Elisabeth H. Bos, Benjamin Hankin, Judith G.M. Rosmalen, Albertine J. Oldehinkel
11. Effects of relationship education on maintenance of couple relationship satisfaction; W. Kim Halford, Guy Bodenmann

12. Psychotherapy for military-related posttraumatic stress disorder: Review of the evidence; Maria M. Steenkamp, Brett T. Litz
13. Sleep disturbance and cognitive deficits in bipolar disorder: Toward an integrated examination of disorder maintenance and functional impairment Elaine M. Boland, Lauren B. Alloy
14. A systematic review of internet-based s elf-help therapeutic interventions to improve distress and disease-control among adults with chronic health conditions; Lisa Beatty, Sylvie Lambert
15. The relationship between anorexia nervosa and body dysmorphic disorder; Andrea S. Hartmann, Jennifer L. Greenberg, Sabine Wilhelm
16. Attention-deficit/hyperactivity disorder and adverse health outcomes; Joel T. Nigg
17. Does neurocognitive functioning predict future or persistence of ADHD? A systematic review M. van Lieshout, M. Luman, J. Buitelaar, N.N.J. Rommelse, J. Oosterlaan
18. Mindfulness: Top down or bottom up emotion regulation strategy? Alberto Chiesa, Alessandro Serretti, Janus Christian Jakobsen
19. Couple and family involvement in adult mental health treatment: A systematic review; Laura A. Meis, Joan M. Griffina Nancy Greer, Agnes C. Jensen, Roderick MacDonald, Maureen Carlyle, Indulis Rutks, Timothy J. Wilt
20. Behavioral parenting interventions for child disruptive behaviors and anxiety: What's different and what's the same; Rex Forehand, Deborah J. Jones, Justin Parent
21. What are the associations between parenting, callous unemotional traits, and antisocial behavior in youth? A systematic review of evidence; Rebecca Waller, Frances Gardner, Luke W. Hyde
22. Specificity of psychological treatments for bulimia nervosa and binge eating disorder? A meta-analysis of direct comparisons; Glen I. Spielmans, Steven G. Benish, Catherine Marin, Wesley M. Bowman, Maria Menster, Anthony J. Wheeler
23. The impact of incarceration on juvenile offenders; Ian Lambie, Isabel Randell
24. A meta-analytic review on treatment dropout in child and adolescent outpatient mental health care; Anna M. de Haan, Albert E. Boon, Joop T.V.M. de Jong, Machteld Hoeve, Robert R.J.M. Vermeiren
25. Psychological consequences of pediatric burns from a child and family perspective: A review of the empirical literature; Anne Bakker, Koen J.P. Maertens, Maarten J.M. Van Son, Nancy E.E. Van Loey
26. Stress generation: Future directions and clinical implications Richard T. Liu
27. Positive emotion regulation in emotional disorders: A theoretical review Jenna R. Carl, David P. Soskin, Caroline Kerns, David H. Barlow
28. Harnessing innovative technologies to advance children's mental health: Behavioral parent training as an example Deborah J. Jones, Rex Forehand, Jessica Cuellar, Carlye Kincaid, Justin Parent, Nicole Fenton, Nada Goodrum

29. Anxiety and oppositional defiant disorder: A transdiagnostic conceptualization; Maria G Fraire, Thomas H. Ollendick

30. Assessing mental imagery in clinical psychology: A review of imagery measures and a guiding framework; David G. Pearson, Catherine Deeprose , Sophie M.A. Wallace-Hadrill, Stephanie Burnett Heyes , Emily A. Holmes

31. Mechanisms of change in interpersonal therapy (IPT); Joshua D. Lipsitz, John C. Markowitz

32. Optimal attentional focus during exposure in specificphobia: Ameta-analysis; Ioana R. Podin, Ernst H.W. Koster, Pierre Philippot, Vincent Dethier, ,DanielO.David

33. The biomedical model of mental disorder: A critical analysis of its validity, utility, and effects on psychotherapy research; Brett J. Deacon

34. The DSM-5 debate over the bereavement exclusion: Psychiatric diagnosis and the future of empirically supported treatment; Jerome C. Wakefield

35. Understanding the impact of political violence in childhood: A theoretical review using a social identity approach; Orla T. Muldoon

36. A systematic review of predictors and moderators of response to psychological therapies in OCD: Do we have enough empirical evidence to target treatment? Jasmin Knopp, Sarah Knowles, Penny Bee, Karina Lovell, Peter Bowe

37. Why many clinical psychologists are resistant to evidence-based practice: Root causes and constructive remedies; Scott O. Lilienfeld, Lorie A. Ritschel, Steven Jay Lynn, Robin L. Cautin, Robert D. Latzman

38. The evidence-based practice of psychotherapy: Facing the challenges that lie ahead; Brandon A. Gaudiano , Ivan W. Miller

39. Health anxiety disorders in older adults: Conceptualizing complex conditions in late life; R. El-Gabalawy, C.S. Mackenzie, M.A. Thibodeau, G.J.G. Asmundson, J. Sareen

40. Death anxiety and its role in psychopathology Reviewing the status of a transdiagnostic construct. L. Iverach

41. The effectiveness of evidence-based treatments for personality disorders when comparing treatment-as-usual and bona fide treatments; Stephanie L. Budge, , Jonathan T. Moore ,A.C.DelRe, Bruce E. Wampold, Timothy P. Baardseth,Jacob B. Nienhuis

42. Acceptance and Commitment Therapy in the treatment of anxiety: A systematic review Jessica Swain, Karen Hancock, Cassandra Hainsworth,JennyBowman

43. Assessing treatments used to reduce rumination and/or worry: A systematic review; Dawn Querstret, MarkCropley

44. Drop-out from addiction treatment: A systematic review of risk factors; Hanne H. Brorson,EspenAjoArnevik, Kim Rand-Hendriksen, Fanny Duckert

45. Systematic information processing style and perseverative worry Suzanne R Dash, Frances Meeten, Graham C L Davey

46. The TEACCH program for children and adults with autism: A meta-analysis of intervention studies, Javier Virues-Ortegaa, Flvia M. Juliob, Roberto Pastor-Barriusoc

47. Lead and Attention-Deficit/Hyperactivity Disorder (ADHD) symptoms: A meta-analysis; James K. Goodlad, David K. Marcus, Jessica J. Fulton

48. Media effects of experimental presentation of the ideal physique on eating disorder symptoms: A meta-analysis of laboratory studies; Heather A. Hausenblas, Anna Campbell, Jessie E. Menzel, Jessica Doughty, Michael Levine, J. Kevin Thompson

49. A meta-analysis of nonrandomized effectiveness studies on outpatient cognitive behavioral therapy for adult anxiety disorders; Eva Hans, Wolfgang Hiller

50. Researcher allegiance in psychotherapy outcome research: An overview of reviews;Thomas Munder, Oliver Brtsch, Rainer Leonhart, Heike Gerger, Jrgen Barth

51. Spanking, corporal punishment and negative long-term outcomes: A meta-analytic review of longitudinal studies; Christopher J. Ferguson

52. Preventing postpartum depression: A meta-analytic review; Laura E. Sockol, C. Neill Epperson, Jacques P. Barber

53. A meta-analysis of cognitive therapy for worry in generalized anxiety disorder; Fidelma Hanrahan, Andy P. Field, Fergal W. Jones, Graham C.L. Davey

54. Impact of support on the effectiveness of written cognitive behavioural self-help: A systematic review and meta-analysis of randomised controlled trials; Paul Farrand, Joanne Woodford

55. Efficacy of psychological interventions aiming to reduce chronic nightmares: A meta-analysis; Kathrin Hansen, Volkmar Hfling, Tana Krner-Borowik, Ulrich Stangier, Regina Steil

56. A meta-analytic review of exposure in group cognitive behavioral therapy for posttraumatic stress disorder; T.L. Barrera, J.M. Mott, R.F. Hofstein, E.J. Teng

57. Aftercare programs for reducing recidivism among juvenile and young adult offenders: A meta-analytic review; Chrissy James, Geert Jan J.M. Stams, Jessica J. Asscher, Anne Katrien De Roo, Peter H. van der Laan

58. Treatment of internet addiction: A meta-analysis Alexander Winkler, Beate Drsing, Winfried Rief, Yuhui Shen, Julia A. Glombiewski

59. Peer similarity and influence for weight-related outcomes in adolescence: A meta-analytic review; Daryaneh Badaly

60. Reaction time variability in ADHD: A meta-analytic review of 319 studies Michael J. Koflera, Mark D. Rapport, Dustin E. Sarver, Joseph S. Raiker, Sarah A. Orban, Lauren M. Friedman, Ellen G. Kolomeyer

61. Mindfulness-based therapy: A comprehensive meta-analysis; Bassam Khoury, Tania Lecomte, Guillaume Fortin, Marjolaine Masse, Phillip Therien, Vanessa Bouchard, Marie-Andre Chapleau, Karine Paquin, Stefan G. Hofmann

62. Efficacy of hypnosis in adults undergoing surgery or medical procedures: A meta-analysis of randomized controlled trials S. Tefikow, J. Barth, S. Maichrowitz, A. Beelmann, B. Strauss, J. Rosendahl

63. Schema therapy for borderline personality disorder: A comprehensive review of its empirical foundations, effectiveness and implementation possibilities; Gabriela A. Semprtegui, Annemiek Karreman, Arnoud Arntz, Marrie H.J. Bekker

64. Toward a consensus definition of pathological video-gaming: A systematic review of psychometric assessment tools Daniel L. King, Maria C. Haagsma, Paul H. Delfabbro, Michael Gradisar, Mark D. Griffiths

65. The side effects of medicalization: A meta-analytic review of how biogenetic explanations affect stigma Erlend P. Kvaale, Nick Haslam, William H. Gottdiener

66. Alternative methods of classifying eat ing disorders: Models incorporating comorbid psychopathology and associated features; Jennifer E. Wildes, Marsha D. Marcus

67. PTSD symptoms associated with the experiences of psychosis and hospitalisation: A review of the literature; Katherine Berry, Sarah Ford,Lorna Jellicoe-Jones, Gillian Haddock

68. Prevalence of child sexual abuse among adults and youths with bipolar disorder: A systematic review; Roberto Maniglio

69. A systematic review of methods for assessing competence in cognitive -behavioural therapy; Kate Muse, Freda McManus

70. The role of impulsivity in pediatric obesity and weight status: A meta-analytic review; Sneha Thamotharan, Krista Lange, Emily L. Zale, Lindsay Huffhines, Sherecce Fields

71. The role of interpersonal functioning in the maintenance of eating psychopathology: A systematic review and testable model Jon Arcelus, Michelle Haslam, Claire Farrow, Caroline Meyer

72. A multi-sample confirmatory factor analysis of PTSD symptoms: What exactly is wrong with the DSM-IV structure? Grant N. Marshall, Terry L. Schell, Jeremy N.V. Miles

73. Adult separation anxiety disorder in DSM-5; Susan M. Bgels, Susanne Knappe, Lee Anna Clark

74. Attachment in old age: Theoretical assumptions, empirical findings and implications for clinical practice Lies Van Assche, Patrick Luyten, Ronny Bruffaerts, Philippe Persoons, Lucas van de Ven, Mathieu Vandenbulcke

75. Emotion regulation and other psychological models for body-focused repetitive behaviors Sarah Roberts, Kieron O'Connor, Claude Blanger

76. Sibling relationship quality and psychopathology of children and adolescents: A meta-analysis Kirsten L. Buist, Maja Dekovic, Peter Prinzie

77. Suicidal behaviour in adolescents and young adults with ASD: Findings from a systematic review Geraldine Hannon, Emily P. Taylor

78. The neuropsychology of adult obsessive compulsive disorder: A meta-analysis; Amitai Abramovitch, Jonathan S. Abramowitz, Andrew Mittelman

79. Instruments for measuring mental health recovery: A systematic review; Marisa Sklar ,ErikJ.Groessl , Maria O'Connell, Larry Davidson, Gregory A. Aarons

80. Impulsivity-related personality traits and adolescent alcohol use: A meta-analytic review Kaidy Stautz, Andrew Cooper

Journal of Consulting and Clinical Psychology

1. Shape of change in cognitive behavioral therapy for youth anxiety: Symptom trajectory and predictors of change. By: Chu, Brian C., Skriner, Laura C., Zandberg, Laurie J.

2. Psychological processes and repeat suicidal behavior: A four-year prospective study. By: OConnor, Rory C., Smyth, Roger, Ferguson, Eamonn, Ryan, Caoimhe, Williams, J. Mark G.

3. Mechanisms of change in an exposure-based treatment for irritable bowel syndrome. By: Ljtsson, Brjnn, Hesser, Hugo, Andersson, Erik, Lindfors, Perjohan, Hursti, Timo, Rck, Christian, Lindefors, Nils, Andersson, Gerhard, Hedman, Erik

4. An adaptive approach for identifying cocaine dependent patients who benefit from extended continuing care. By: McKay, James R., Van Horn, Deborah H. A., Lynch, Kevin G., Ivey, Megan, Cary, Mark S., Drapkin, Michelle L., Coviello, Donna M., Plebani, Jennifer G.

5. Two-year course of generalized anxiety disorder, social anxiety disorder, and panic disorder in a longitudinal sample of African American adults. By: Sibrava, Nicholas J., Beard, Courtney, Bjornsson, Andri S., Moitra, Ethan, Weisberg, Risa B., Keller, Martin B

6. Multilevel context of depression in two American Indian tribes. By: Kaufman, Carol E., Beals, Janette, Croy, Calvin, Jiang, Luohua, Novins, Douglas K.

7. An independent randomized clinical trial of multisystemic therapy with non-court-referred adolescents with serious conduct problems. By: Weiss, Bahr, Han, Susan, Harris, Vicki, Catron, Thomas, Ngo, Victoria K., Caron, Annalise, Gallop, Robert, Guth, Carol

8. Randomized controlled trial of parent-enhanced CBT compared with individual CBT for obsessive-compulsive disorder in young people. By: Reynolds, Shirley A., Clark, Sarah, Smith, Holly, Langdon, Peter E., Payne, Ruth, Bowers, Gemma, Norton, Elisabeth, McIlwham, Harriet

9. Working alliance and treatment fidelity as predictors of externalizing problem behaviors in parent management training. By: Hukkelberg, Silje S., Ogden, Terje

10. Long-term outcomes for the Child STEPs randomized effectiveness trial: A comparison of modular and standard treatment designs with usual care. By: Chorpita, Bruce F., Weisz, John R., Daleiden, Eric L., Schoenwald, Sonja K., Palinkas, Lawrence A., Miranda, Jeanne, Higa-McMillan, Charmaine K., Nakamura, Brad J., Austin, A. Aukahi, Borntrager, Cameo F., Ward, Alyssa, Wells, Karen C., Gibbons, Robert D

11. School-based intervention for childhood disruptive behavior in disadvantaged settings: A randomized controlled trial with and without active teacher support. By: Liber, Juliette M., De Boo, Gerly M., Huizenga, Hilde, Prins, Pier J. M.

12. Indirect effects of fidelity to the family check-up on changes in parenting and early childhood problem behaviors. By: Smith, Justin D., Dishion, Thomas J., Shaw, Daniel S., Wilson, Melvin N

13. Is skills training necessary for the primary prevention of marital distress and dissolution? A 3-year experimental study of three interventions. By: Rogge, Ronald D., Cobb, Rebecca J., Lawrence, Erika, Johnson, Matthew D., Bradbury, Thomas N

14. The efficacy of a family-based cognitive-behavioral treatment for separation anxiety disorder in children aged 813: A randomized comparison with a general anxiety program. By: Schneider, Silvia, Blatter-Meunier, Judith, Herren, Chantal, In-Albon, Tina, Adornetto, Carmen, Meyer, Andrea, Lavallee, Kristen L.

15. Development and preliminary evaluation of an integrated treatment targeting parenting and depressive symptoms in mothers of children with attention-deficit/hyperactivity disorder. By: Chronis-Tuscano, Andrea, Clarke, Tana L., OBrien, Kelly A., Raggi, Veronica L., Diaz, Yamalis, Mintz, Abigail D., Rooney, Mary E., Knight, Laura A., Seymour, Karen E., Thomas, Sharon R., Seeley, John, Kosty, Derek, Lewinsohn, Peter

16. National dissemination of cognitive behavioral therapy for insomnia in veterans: Therapist- and patient-level outcomes. By: Karlin, Bradley E., Trockel, Mickey, Taylor, C. Barr, Gimeno, Julia, Manber, Rachel

17. The immigrant paradox among Asian American women: Are disparities in the burden of depression and anxiety paradoxical or explicable? By: Lau, Anna S., Tsai, William, Shih, Josephine, Liu, Lisa L., Hwang, Wei-Chin, Takeuchi, David T.

18. Web intervention for OEF/OIF veterans with problem drinking and PTSD symptoms: A randomized clinical trial. By: Brief, Deborah J., Rubin, Amy, Keane, Terence M., Enggasser, Justin L., Roy, Monica, Helmuth, Eric, Hermos, John, Lachowicz, Mark, Rybin, Denis, Rosenbloom, David

19. Anxiety and related outcomes in young adults 7 to 19 years after receiving treatment for child anxiety. By: Benjamin, Courtney L., Harrison, Julie P., Settipani, Cara A., Brodman, Douglas M., Kendall, Philip C.

20. Narrative meaning making is associated with sudden gains in psychotherapy clients mental health under routine clinical conditions. By: Adler, Jonathan M., Harmeling, Luke H., Walder-Biesanz, Ilana

21. Relationship of childhood trauma to depression and smoking outcomes in pregnant smokers. By: Blalock, Janice A., Minnix, Jennifer A., Mathew, Amanda R., Wetter, David W., McCullough, James P. Jr., Cinciripini, Paul M.

22. Efficacy of an experiential, dissonance-based smoking intervention for college students delivered via the Internet. By: Simmons, Vani Nath, Heckman, Bryan W., Fink, Angelina C., Small, Brent J., Brandon, Thomas H.

23. Combining imagination and reason in the treatment of depression: A randomized controlled trial of internet-based cognitive-bias modification and internet-CBT for depression. By: Williams, Alishia D., Blackwell, Simon E., Mackenzie, Anna, Holmes, Emily A., Andrews, Gavin

24. Detecting critical decision points in psychotherapy and psychotherapy + medication for chronic depression. By: Steidtmann, Dana, Manber, Rachel, Blasey, Christine, Markowitz, John C., Klein, Daniel N., Rothbaum, Barbara O., Thase, Michael E., Kocsis, James H., Arnow, Bruce A.

25. Pretreatment anxiety predicts patterns of change in cognitive behavioral therapy and medications for depression. By: Forand, Nicholas R., DeRubeis, Robert J.

26. A randomized clinical trial comparing an acceptance-based behavior therapy to applied relaxation for generalized anxiety disorder. By: Hayes-Skelton, Sarah A., Roemer, Lizabeth, Orsillo, Susan M.

27. Virtual reality exposure therapy for social anxiety disorder: A randomized controlled trial. By: Anderson, Page L., Price, Matthew, Edwards, Shannan M., Obasaju, Mayowa A., Schmertz, Stefan K., Zimand, Elana, Calamaras, Martha R.

28. Emotional congruence with children and sexual offending against children: A meta-analytic review. By: McPhail, Ian V., Hermann, Chantal A., Nunes, Kevin L.

29. Are the parts as good as the whole? A meta-analysis of component treatment studies. By: Bell, Erin C., Marcus, David K., Goodlad, James K.

30. Race/ethnicity, education, and treatment parameters as moderators and predictors of outcome in binge eating disorder. By: Thompson-Brenner, Heather, Franko, Debra L., Thompson, Douglas R., Grilo, Carlos M., Boisseau, Christina L., Roehrig, James P., Richards, Lauren K., Bryson, Susan W., Bulik, Cynthia M., Crow, Scott J., Devlin, Michael J., Gorin, Amy A., Kristeller, Jean L., Masheb, Robin, Mitchell, James E., Peterson, Carol B., Safer, Debra L., Striegel, Ruth H., Wilfley, Denise E., Wilson, G. Terence

31. The efficacy of single-component brief motivational interventions among at-risk college drinkers. By: Martens, Matthew P., Smith, Ashley E., Murphy, James G.

32. Comparison of youth, caregiver, therapist, trained, and treatment expert raters of therapist adherence to a substance abuse treatment protocol. By: Chapman, Jason E., McCart, Michael R., Letourneau, Elizabeth J., Sheidow, Ashli J.

33. Fifteen-year follow-up of a randomized trial of a preventive intervention for divorced families: Effects on mental health and substance use outcomes in young adulthood. By: Wolchik, Sharlene A., Sandler, Irwin N., Tein, Jenn-Yun, Mahrer, Nicole E., Millsap, Roger E., Winslow, Emily, Vlez, Clorinda, Porter, Michele M., Luecken, Linda J., Reed, Amanda

34. The relationship between the therapeutic alliance and treatment outcome in two distinct psychotherapies for chronic depression. By: Arnow, Bruce A., Steidtmann, Dana, Blasey, Christine, Manber, Rachel, Constantino, Michael J., Klein, Daniel N., Markowitz, John C., Rothbaum, Barbara O., Thase, Michael E., Fisher, Aaron J., Kocsis, James H.

35. Intervention with substance-abusing runaway adolescents and their families: Results of a randomized clinical trial. By: Slesnick, Natasha, Erdem, Gizem, Bartle-Haring, Suzanne, Brigham, Gregory S.

36. A randomized controlled trial of COMPASS web-based and face-to-face teacher coaching in autism. By: Ruble, Lisa A., McGrew, John H., Toland, Michael D., Dalrymple, Nancy J., Jung, Lee Ann

37. Specificity and modifiability of cognitive biases in hypochondriasis. By: Gropalis, Maria, Bleichhardt, Gaby, Hiller, Wolfgang, Witthft, Michael

38. Developing a method for specifying the components of behavior change interventions in practice: The example of smoking cessation. By: Lorencatto, Fabiana, West, Robert, Seymour, Natalie, Michie, Susan

39. Latent profile analysis to determine the typology of disinhibited eating behaviors in children and adolescents. By: Vannucci, Anna, Tanofsky-Kraff, Marian, Crosby, Ross D., Ranzenhofer, Lisa M., Shomaker, Lauren B., Field, Sara E., Mooreville, Mira, Reina, Samantha A., Kozlosky, Merel, Yanovski, Susan Z., Yanovski, Jack A.

40. Sympathetic- and parasympathetic-linked cardiac function and prediction of externalizing behavior, emotion regulation, and prosocial behavior among preschoolers treated for ADHD. By: Beauchaine, Theodore P., Gatzke-Kopp, Lisa, Neuhaus, Emily, Chipman, Jane, Reid, M. Jamila, Webster-Stratton, Carolyn

41. Patient characteristics and variability in adherence and competence in cognitive-behavioral therapy for panic disorder. By: Boswell, James F., Gallagher, Matthew W., Sauer-Zavala, Shannon E., Bullis, Jacqueline, Gorman, Jack M., Shear, M. Katherine, Woods, Scott, Barlow, David H.

42. Hybrid cognitive behavioral therapy versus relaxation training for co-occurring anxiety and alcohol disorder: A randomized clinical trial. By: Kushner, Matt G., Maurer, Eric W., Thuras, Paul, Donahue, Chris, Frye, Brenda, Menary, Kyle R., Hobbs, Jennifer, Haeny, Angela M., Van Demark, Joani

43. Behavioral versus cognitive treatment of obsessive-compulsive disorder: An examination of outcome and mediators of change. By: Olatunji, Bunmi O., Rosenfield, David, Tart, Candyce D., Cottraux, Jean, Powers, Mark B., Smits, Jasper A. J.

44. Meta-analysis of dropout in treatments for posttraumatic stress disorder. By: Imel, Zac E., Laska, Kevin, Jakupcak, Matthew, Simpson, Tracy L

45. Cognitive change predicts symptom reduction with cognitive therapy for posttraumatic stress disorder. By: Kleim, Birgit, Grey, Nick, Wild, Jennifer, Nussbeck, Fridtjof W., Stott, Richard, Hackmann, Ann, Clark, David M., Ehlers, Anke

46. The relationship between posttraumatic and depressive symptoms during prolonged exposure with and without cognitive restructuring for the treatment of posttraumatic stress disorder. By: Aderka, Idan M., Gillihan, Seth J., McLean, Carmen P., Foa, Edna B.

47. Chronic hepatitis C and antiviral treatment regimens: Where can psychology contribute? By: Evon, Donna M., Golin, Carol E., Fried, Michael W., Keefe, Francis J.

48. Treatment of childhood and adolescent obesity: An integrative review of recent recommendations from five expert groups. By: Kirschenbaum, Daniel S., Gierut, Kristen

49. Promoting healthy weight with stability skills first: A randomized trial. By: Kiernan, Michaela, Brown, Susan D., Schoffman, Danielle E., Lee, Katherine, King, Abby C., Taylor, C. Barr, Schleicher, Nina C., Perri, Michael G.

50. Mechanisms in psychosocial interventions for adults living with cancer: Opportunity for integration of theory, research, and practice. By: Stanton, Annette L., Luecken, Linda J., MacKinnon, David P., Thompson, Elizabeth H.

51. Gender-specific effects of an augmented written emotional disclosure intervention on post-traumatic, depressive, and HIV-disease-related outcomes: A randomized, controlled trial. By: Ironson, Gail, O'Cleirigh, Conall, Leserman, Jane, Stuetzle, Rick, Fordiani, Joanne, Fletcher, MaryAnn, Schneiderman, Neil

52. Reductions in traumatic stress following a coping intervention were mediated by decreases in avoidant coping for people living with HIV/AIDS and childhood sexual abuse. By: Sikkema, Kathleen J., Ranby, Krista W., Meade, Christina S., Hansen, Nathan B., Wilson, Patrick A., Kochman, Arlene

53. Treating depressed and anxious smokers in smoking cessation programs. By: Richards, C. Steven, Cohen, Lee M., Morrell, Holly E. R., Watson, Noreen L., Low, Blakely E.

54. A randomized controlled trial of cognitive behavioral therapy (CBT) for adjusting to multiple sclerosis (the saMS trial): Does CBT work and for whom does it work? By: Moss-Morris, Rona, Dennison, Laura, Landau, Sabine, Yardley, Lucy, Silber, Eli, Chalder, Trudie

55. Psychosocial factors and behavioral medicine interventions in asthma. By: Ritz, Thomas, Meuret, Alicia E., Trueba, Ana F., Fritzsche, Anja, von Leupoldt, Andreas

56. Translating basic behavioral and social science research to clinical application: The EVOLVE mixed methods approach. By: Peterson, Janey C., Czajkowski, Susan, Charlson, Mary E., Link, Alissa R., Wells, Martin T., Isen, Alice M., Mancuso, Carol A., Allegrante, John P., Boutin-Foster, Carla, Ogedegbe, Gbenga, Jobe, Jared B.

57. Religiousness/spirituality, cardiovascular disease, and cancer: Cultural integration for health research and intervention. By: Masters, Kevin S., Hooker, Stephanie A.

58. Efficacy trial of a selective prevention program targeting both eating disorders and obesity among female college students: 1- and 2-year follow-up effects. By: Stice, Eric, Rohde, Paul, Shaw, Heather, Marti, C. Nathan

59. Sudden gains in cognitive therapy and interpersonal therapy for social anxiety disorder. By: Bohn, Christiane, Aderka, Idan M., Schreiber, Franziska, Stangier, Ulrich, Hofmann, Stefan G.

60. Biological sensitivity to context in couples: Why partner aggression hurts some more than others. By: Lorber, Michael F., Erlanger, Ann C. Eckardt, Slep, Amy M. Smith

61. Patient-rated alliance as a measure of therapist performance in two clinical settings. By: Imel, Zac E., Hubbard, Rebecca A., Rutter, Carolyn M., Simon, Gregory

62. Multi-tier mental health program for refugee youth. By: Ellis, B. Heidi, Miller, Alisa B., Abdi, Saida, Barrett, Colleen, Blood, Emily A., Betancourt, Theresa S

63. Remediating organizational functioning in children with ADHD: Immediate and long-term effects from a randomized controlled trial. By: Abikoff, Howard, Gallagher, Richard, Wells, Karen C., Murray, Desiree W., Huang, Lei, Lu, Feihan, Petkova, Eva

64. A randomized trial of a classroom intervention to increase peers' social inclusion of children with attention-deficit/hyperactivity disorder. By: Mikami, Amori Yee, Griggs, Marissa Swaim, Lerner, Matthew D., Emeh, Christina C., Reuland, Meg M., Jack, Allison, Anthony, Maria R.

65. Effectiveness of and dropout from outpatient cognitive behavioral therapy for adult unipolar depression: A meta-analysis of nonrandomized effectiveness studies. By: Hans, Eva, Hiller, Wolfgang

66. Publication bias in meta-analyses of the efficacy of psychotherapeutic interventions for depression. By: Niemeyer, Helen, Musch, Jochen, Pietrowsky, Reinhard

67. Implicit and Explicit Drug-Related Cognitions During Detoxification Treatment Are Associated With Drug Relapse: An Ecological Momentary Assessment Study; MARHE, WATERS, VAN DE WETERING, AND FRANKEN

68. The Effects of Drinking Goal on Treatment Outcome for Alcoholism; BUJARSKI, OMALLEY, LUNNY, AND RAY

69. Process Predictors of the Outcome of Group Drug Counseling; CRITS-CHRISTOPH, JOHNSON, CONNOLLY GIBBONS, AND GALLOP

2 Rejection of linear regression on basis of correct assumptions

Journal of Consulting and Clinical Psychology

1. RCT of web-based personalized normative feedback for college drinking prevention: Are typical student norms good enough? By: LaBrie, Joseph W., Lewis, Melissa A., Atkins, David C., Neighbors, Clayton, Zheng, Cheng, Kenney, Shannon R., Napper, Lucy E., Walter, Theresa, Kilmer, Jason R., Hummer, Justin F., Grossbard, Joel, Ghaidarov, Tehniat M., Desai, Sruti, Lee, Christine M., Larimer, Mary E.

2. Indicated prevention for college student marijuana use: A randomized controlled trial. By: Lee, Christine M., Kilmer, Jason R., Neighbors, Clayton, Atkins, David C., Zheng, Cheng, Walker, Denise D., Larimer, Mary E.

3. The temporal association between substance use and intimate partner violence among women arrested for domestic violence. By: Stuart, Gregory L., Moore, Todd M., Elkins, Sara R., OFarrell, Timothy J., Temple, Jeff R., Ramsey, Susan E., Shorey, Ryan C.

3 Rejection of linear regression on basis of not meeting incorrect assumptions

4 Correct linear regression

5 Mentioned all correct assumptions but not if the normality assumption was tested on the residuals or on X or Y

6 Did not test all but some correct assumptions, included neither normality of variables nor residuals

Clinical Psychology Review

1. Offspring psychological and biological correlates of parental posttraumatic stress: Review of the literature and research agenda; Ellen W. Leen-Feldner, Matthew T. Feldner, Ashley Knapp, Liviu Bunaciu, Heidemarie Blumenthal,Ananda B. Amstadter

2. Do programs designed to train working memory, other executive functions, and attention benefit children with ADHD? A meta-analytic review of cognitive, academic, and behavioral outcomes; Mark D. Rapport, Sarah A. Orban, MichaelJ.Kofler, Lauren M. Friedman

Journal of Consulting and Clinical Psychology

1. D-cycloserine augmentation of cognitive behavioral group therapy of social anxiety disorder: Prognostic and prescriptive variables. By: Smits, Jasper A. J., Hofmann, Stefan G., Rosenfield, David, DeBoer, Lindsey B., Costa, Paul T., Simon, Naomi M., OCleirigh, Conall, Meuret, Alicia E., Marques, Luana, Otto, Michael W., Pollack, Mark H.

7 Use of linear regression but no indication if any or which assumptions were tested

Clinical Psychology Review

1. Life events and treatment outcomes among individuals with substance use disorders: A narrative review; Marketa Krenek, Stephen A. Maisto

2. Conditional risk for PTSD among Latinos: A systematic review of racial/ethnic differences and sociocultural explanations Carmela Alcntara, Melynda D. Casement, Roberto Lewis-Fernndez

3. Use of alcohol protective behavioral strategies among college students: A critical review, M.R. Pearson

4. Emotion processing in Psychopathy Checklist assessed psychopathy: A review of the literature; Michael Brook , Chelsea L. Brieman , David S. Kosson

Journal of Consulting and Clinical Psychology

1. Nomothetic and idiographic symptom change trajectories in acute-phase cognitive therapy for recurrent depression. By: Vittengl, Jeffrey R., Clark, Lee Anna, Thase, Michael E., Jarrett, Robin B.

2. Therapist-aided exposure for women with lifelong vaginismus: A randomized waiting-list control trial of efficacy. By: ter Kuile, Moniek M., Melles, Reinhilde, de Groot, H. Ellen, Tuijnman-Raasveld, Charlotte C., van Lankveld, Jacques J. D. M.

3. Alcohol craving in patients diagnosed with a severe mental illness and alcohol use disorder: Bidirectional relationships between approach and avoidance inclinations and drinking. By: Schlauch, Robert C., Levitt, Ash, Bradizza, Clara M., Stasiewicz, Paul R., Lucke, Joseph F., Maisto, Stephen A., Zhuo, Yue, Connors, Gerard J.

4. Parental ADHD symptoms and self-reports of positive parenting. By: Lui, Joyce H. L., Johnston, Charlotte, Lee, Catherine M., Lee-Flynn, Sharon C

5. Contingency management treatments decrease psychiatric symptoms. By: Petry, Nancy M., Alessi, Sheila M., Rash, Carla J.

6. Posttraumatic stress and posttraumatic growth among low-income mothers who survived Hurricane Katrina. By: Lowe, Sarah R., Manove, Emily E., Rhodes, Jean E.

7. The therapeutic relationship in cognitive-behavioral therapy and pharmacotherapy for anxious youth. By: Cummings, Colleen M., Caporino, Nicole E., Settipani, Cara A., Read, Kendra L., Compton, Scott N., March, John, Sherrill, Joel, Piacentini, John, McCracken, James, Walkup, John T., Ginsburg, Golda, Albano, Anne Marie, Rynn, Moira, Birmaher, Boris, Sakolsky, Dara, Gosch, Elizabeth, Keeton, Courtney, Kendall, Philip C.

8. Does childhood positive self-perceptual bias mediate adolescent risky behavior in youth from the MTA study? By: Hoza, Betsy, McQuade, Julia D., Murray-Close, Dianna, Shoulberg, Erin, Molina, Brooke S. G., Arnold, L. Eugene, Swanson, James, Hechtman, Lily

9. Mediators of the effects on fatigue of pragmatic rehabilitation for chronic fatigue syndrome. By: Wearden, Alison J., Emsley, Richard

10. The effect of placebo tailoring on smoking cessation: A randomized controlled trial. By: Webb Hooper, Monica, Rodrguez de Ybarra, Denise, Baker, Elizabeth A.

11. Chronic sleep disturbances and borderline personality disorder symptoms. By: Selby, Edward A.

12. Convergent and incremental predictive validity of clinician, self-report, and structured interview diagnoses for personality disorders over 5 years. By: Samuel, Douglas B., Sanislow, Charles A., Hopwood, Christopher J., Shea, M. Tracie, Skodol, Andrew E., Morey, Leslie C., Ansell, Emily B., Markowitz, John C., Zanarini, Mary C., Grilo, Carlos M.

13. Who gets the most out of cognitive behavioral therapy for anxiety disorders? The role of treatment dose and patient engagement. By: Glenn, Daniel, Golinelli, Daniela, Rose, Raphael D., Roy-Byrne, Peter, Stein, Murray B., Sullivan, Greer, Bystritksy, Alexander, Sherbourne, Cathy, Craske, Michelle G.

14. The predictive utility of a brief kindergarten screening measure of child behavior problems. By: Racz, Sarah Jensen, King, Kevin M., Wu, Johnny, Witkiewitz, Katie, McMahon, Robert J.

15. Anxiety and depression in transgender individuals: The roles of transition status, loss, social support, and coping. By: Budge, Stephanie L., Adelson, Jill L., Howard, Kimberly A. S.

16. Fixable or fate? Perceptions of the biology of depression. By: Lebowitz, Matthew S., Ahn, Woo-kyoung, Nolen-Hoeksema, Susan

17. Mood reactivity rather than cognitive reactivity is predictive of depressive relapse: A randomized study with 5.5-year follow-up. By: van Rijsbergen, Gerard D., Bockting, Claudi L. H., Burger, Huibert, Spinhoven, Philip, Koeter, Maarten W. J., Ruh, Henricus G., Hollon, Steven D., Schene, Aart H.

18. Psychopathic predators? Getting specific about the relation between psychopathy and violence. By: Camp, Jacqueline P., Skeem, Jennifer L., Barchard, Kimberly, Lilienfeld, Scott O., Poythress, Norman G.

19. Dimensions of functional social support and depressive symptoms: A longitudinal investigation of women seeking help for intimate partner violence. By: Suvak, Michael K., Taft, Casey T., Goodman, Lisa A., Dutton, Mary Ann

20. Mediated moderation in combined cognitive behavioral therapy versus component treatments for generalized anxiety disorder. By: Newman, Michelle G., Fisher, Aaron J.

21. Psychological factors associated with head and neck cancer treatment and survivorship: Evidence and opportunities for behavioral medicine. By: Howren, M. Bryant, Christensen, Alan J., Karnell, Lucy Hynds, Funk, Gerry F

22. Disclosure and concealment of sexual orientation and the mental health of non-gay-identified, behaviorally bisexual men. By: Schrimshaw, Eric W., Siegel, Karolynn, Downing, Martin J. Jr., Parsons, Jeffrey T.

23. Telephone-based physical activity counseling for major depression in people with multiple sclerosis. By: Bombardier, Charles H., Ehde, Dawn M., Gibbons, Laura E., Wadhwani, Roini, Sullivan, Mark D., Rosenberg, Dori E., Kraft, George H

24. Does maintenance CBT contribute to long-term treatment response of panic disorder with or without agoraphobia? A randomized controlled clinical trial. By: White, Kamila S., Payne, Laura A., Gorman, Jack M., Shear, M. Katherine, Woods, Scott W., Saksa, John R., Barlow, David H.

25. Effect of a significant other on client change talk in motivational interviewing. By: Apodaca, Timothy R., Magill, Molly, Longabaugh, Richard, Jackson, Kristina M., Monti, Peter M.,

8 Assumed/tested normally distributed X but not the normality of the residuals

9 Assumed/tested normally distributed Y but not the normality of the residuals

10 Assumed/tested normally distributed X and Y but not the normality of the residuals

11 Assumed/tested normally distributed variables but did not indicate if X or Y or both and did not test the normality of the residuals

12 Other misconceptions about assumptions

Part II
Q2 top 3 Clinical Journals: International Psychogeriatrics, Journal of Attention Disorders, American Journal of Drug and Alcohol Abuse

1 No Model of Interest

International Psychogeriatrics

1. Assessing mental well-being in family carers of people with dementia using the WarwickEdinburgh Mental Well-Being Scale; Vasiliki Orgeta, Elena Lo Sterzo and Martin Orrell
2. Peritraumatic distress but not dissociation predicts posttraumatic stress disorder in the elderly; Alain Brunet, Steven Sanche, Aude Manetti, Bruno Aouizerate, Rgis Ribreau-Gayon, Sandrine Charpentier, Philippe Birmes and Christophe Arbus
3. Addressing elder abuse: review of societal responses in India and selected Asian countries; Mala Kapur Shankardass
4. The effect of exercise interventions on cognitive outcome in Alzheimers disease: a systematic review; Nicolas Farina, Jennifer Rusted and Naji Tabet
5. Detection of mild cognitive impairment and early stage dementia with an audio-recorded cognitive scale; Margaret C. Sewell, Xiaodong Luo, Judith Neugroschl and Mary Sano

6. The relationship between older people's awareness of the term elder abuse and actual experiences of elder abuse ; Corina Naughton, Jonathan Drennan, Imogen Lyons and Attracta Lafferty
7. Critical concepts in elder abuse research; Thomas Goergen and Marie Beaulieu
8. The social construction of social problems: the case of elder abuse and neglect; Zvi Eisikovits, Chaya Koren and Tova Band-Winterstein
9. Predictors of prolonged hospital stay for the treatment of severe neuropsychiatric symptoms in patients with dementia: a cohort study in multiple hospitals; Hiromichi Sugiyama, Hiroaki Kazui, Kazue Shigenobu, Yoshihiro Masaki, Naoki Hatta, Daisuke Yamamoto, Tamiki Wada, Keiko Nomura, Kenji Yoshiyama, Kaoru Tabushi and Masatoshi Takeda
10. Acute dystonic reaction with rivastigmine; Vikas Dhikav and Kuljeet Singh Anand
11. Using qualitative methods to develop a measure of resident-to-resident elder mistreatment in nursing homes; Mildred Ramirez, Beverly Watkins, Jeanne A. Teresi, Stephanie Silver, Gail Sukha, Gabriel Bortagis, Kimberly Van Haitsma, Mark S. Lachs and Karl Pillemer
12. The dark side of family communication: a communication model of elder abuse and neglect, Mei-Chen Lin and Howard Giles
13. Elder abuse through a life course lens; Lynn McDonald and Cynthia Thomas
14. Elder mistreatment, ageism, and human rights; Simon Biggs and Irja Haapala
15. Framing abuse: explaining the incidence, perpetuation, and intervention in elder abuse; Yuliya Mysyuk, Rudi G. J. Westendorp and Jolanda Lindenberg
16. Neuropsychiatric disorders secondary to neurosyphilis in elderly people: one theme not to be ignored; Yan-Li Zeng, Wen-Jie Wang, Hui-Lin Zhang, Fu-Yi Chen, Song-Jie Huang, Gui-Li Liu, Ya Xi, Xiao-Jing Guo, Wei-Hong Zheng and Tian-Ci Yang
17. Can medication management review reduce anticholinergic burden (ACB) in the elderly? Encouraging results from a theoretical model; Zikai He and Patrick Anthony Ball
18. Association between behavioral and psychological symptoms and psychotropic drug use among old people with cognitive impairment living in geriatric care settings; Maria Gustafsson, Per-Olof Sandman, Stig Karlsson, Yngve Gustafson and Hugo Lvheim
19. Assessment of anxiety in long-term care: examination of the Geriatric Anxiety Inventory (GAI) and its short form; Lindsay A. Gerolimatos, Jeffrey J. Gregg and Barry A. Edelstein
20. Translation and validation of the 18-item Lubben Social Network Scale with older adults in Mongolia; Denise Burnette and Sugarmaa Myagmarjav
21. Do dietary patterns influence cognitive function in old age? Janie Corley, John M. Starr, Geraldine McNeill and Ian J. Deary
22. Membership and management: structures of inter-professional working in community mental health teams for older people in England; Mark Wilberforce, Sue Tucker, Michele Abendstern, Christian Brand, Clarissa Marie Giebel and David Challis

23. Are there sensitive time periods for dementia caregivers? The occurrence of behavioral and psychological symptoms in the early stages of dementia; K. A. Ornstein, J. E. Gaugler, D. P. Devanand, N. Scarmeas, C. W. Zhu and Y. Stern

24. Medication adherence survey of drugs useful in prevention of dementia of Alzheimer's type among Indian patients; Vikas Dhikav, Pritika Singh and Kuljeet Singh Anand

25. Escitalopram for antipsychotic nonresponsive visual hallucinosis: eight patients suffering from Charles Bonnet syndrome; Yoseph Bergmaen, Mikael Fraunberg, Kristiina Hongisto, Tarja Vlimki, Asta Hiltunen, Pertti Karppi, Juhani Sivenius, Hilkka Soininen and Anne M. KoivistoBarak

26. Cost of informal care for community-dwelling mildmoderate dementia patients in a developed Southeast Asian country; Mei Sian Chong, Woan Shin Tan, Mark Chan, Wee Shiong Lim, Noorhazlina Ali, Yue Ying Ang and Kia Chong Chua

27. Sensitivity of cognitive tests in four cognitive domains in discriminating MDD patients from healthy controls: a meta-analysis; JaeHyoung Lim, In Kyung Oh, Changsu Han, Yu Jeong Huh, In-Kwa Jung, Ashwin A. Patkar, David C. Steffens and Bo-Hyoung Jang

28. Family caregiving at the intersection of private care by migrant home care workers and public care by nursing staff ; Liat Ayalon, Sara Halevy-Levin, Zvi Ben-Yizhak and Gideon Friedman

29. Validity and reliability of the PDCB: a tool for the assessment of caregiver burden in Parkinson's disease; Michael Zhong, Andrew Evans, Richard Peppard and Dennis Velakoulis

30. Perceived unmet needs of informal caregivers of people with dementia in Singapore; Janhavi Ajit Vaingankar, Mythily Subramaniam, Louisa Picco, Goi Khia Eng, Saleha Shafie, Rajeswari Sambasivam, Yun Jue Zhang, Vathsala Sagayadevan and Siow Ann Chong

31. One day in the life of old age psychiatrists in the United Kingdom; Susan Mary Benbow and David Jolley

32. Aspects of communication in Alzheimer's disease: clinical features and treatment options Journal; Michael Woodward

33. Everyday decision-making in dementia: findings from a longitudinal interview study of people with dementia and family carer; Kritika Samsi and Jill Manthorpe

34. Establishing the motivations of patients with dementia and cognitive impairment and their carers in joining a dementia research register (DemReg); Cerian Avent, Lisa Curry, Sarah Gregory, Sonia Marquardt, Lauren Pae, Danielle Wilson, Karen Ritchie and Craig W. Ritchie

35. A randomized, double-blind, placebo-controlled trial of memantine in a behaviorally enriched sample of patients with moderate-to-severe Alzheimer's disease; Nathan Herrmann, Serge Gauthier, Neli Boneva and Ole Michael Lemming

36. Symptoms of delirium predict incident delirium in older long-term care residents; Martin G. Cole, Jane McCusker, Philippe Voyer, Johanne Monette, Nathalie Champoux, Antonio Ciampi, Minh Vu, Alina Dyachenko and Eric Belzile

37. The Sydney Centenarian Study: methodology and profile of centenarians and near-centenarians; Perminder S. Sachdev, Charlene Levitan, John Crawford, Mamta Sidhu, Melissa Slavin, Robyn Richmond, Nicole Kochan, Henry Brodaty, Wei Wen, Kristan Kang and Karen A. Mather

38. Alcohol, hospital admissions, and falls in older adults: a longitudinal evaluation; Robert J. Tait, Davina J. French, Richard A. Burns, Julie E. Byles and Kaarin J. Anstey

39. An intermediate care unit for older people with both physical and psychiatric disorders: naturalistic outcome study; Claire Hilton, Andrew Madaras and Maria Qureshi

40. Content of delusional thoughts in Alzheimer's disease and assessment of content-specific brain dysfunctions with BEHAVE-AD-FW and SPECT; Masahiro Nakatsuka, Kenichi Meguro, Hiroshi Tsuboi, Kei Nakamura, Kyoko Akanuma and Satoshi Yamaguchi

41. Healthy behavior and memory self-reports in young, middle-aged, and older adults; Gary W. Small, Prabha Siddarth, Linda M. Ercoli, Stephen T. Chen, David A. Merrill and Fernando Torres-Gil

42. Moving in: adjustment of people living with dementia going into a nursing home and their families; Laura Sury, Kim Burns and Henry Brodaty

43. Venous thromboembolism in psychogeriatric in-patients A study of risk assessment, incidence, and current prophylaxis prescribing; Xinsheng Liu, Fintan O'Rourke and Huong Van Nguyen

44. The usefulness of monitoring sleep talking for the diagnosis of dementia with Lewy bodies; Kazuki Honda, Mamoru Hashimoto, Yusuke Yatabe, Keiichiro Kaneda, Seiji Yuki, Yusuke Ogawa, Shiho Matsuzaki, Atsuko Tsuyuguchi, Hibiki Tanaka, Hiroko Kashiwagi, Noriko Hasegawa, Tomohisa Ishikawa and Manabu Ikeda

45. How do family carers respond to behavioral and psychological symptoms of dementia? Kirsten Moore, Elizabeth Ozanne, David Ames and Briony Dow

46. Efficacy of a cognitive intervention program in patients with mild cognitive impairment; Galeno J. Rojas, Veronica Villar, , Paula Harris, Cecilia M. Serrano, Jorge A. Herrera and Ricardo F. Allegri

47. Treatment fidelity and acceptability of a cognition-focused intervention for older adults with mild cognitive impairment (MCI); Mandy R. Vidovich, Nicola T. Lautenschlager, Leon Flicker, Linda Clare and Osvaldo P. Almeida

48. Exploration of verbal and non-verbal semantic knowledge and autobiographical memories starting from popular songs in Alzheimer's disease; S. Basaglia-Pappas, M. Laterza, C. Borg, A. Richard-Mornas, E. Favre and C. Thomas-Antrion

49. Perspectives of the community-based dementia care workforce: occupational communion a key finding from the Work 4 Dementia Project; Kate-Ellen J. Elliott, Christine M. Stirling, Angela J. Martin, Andrew L. Robinson and Jennifer L. Scott

50. Health-related quality-of-life instruments for Alzheimer's disease and mixed dementia; Jaime Perales, Theodore D. Cosco, Blossom C. M. Stephan, Josep Maria Haro and Carol Brayne

51. Care workers abusive behavior to residents in care homes: a qualitative study of types of abuse, barriers, and facilitators to good care and development of an instrument for reporting of abuse anonymously; Claudia Cooper, Briony Dow, Susan Hay, Deborah Livingston and Gill Livingston

52. Comparing the effects of different individualized music interventions for elderly individuals with severe dementia; Mayumi Sakamoto, Hiroshi Ando and Akimitsu Tsutou

53. 12-month incidence, prevalence, persistence, and treatment of mental disorders among individuals recently admitted to assisted living facilities in Maryland; Quincy M. Samus, Chiadi U. Onyike, Deirdre Johnston, Lawrence Mayer, Matthew McNabney, Alva S. Baker, Jason Brandt, Peter V. Rabins, Constantine G. Lyketsos and Adam Rosenblatt

54. Multi-state Markov model in outcome of mild cognitive impairments among community elderly residents in Mainland China; Hong-mei Yu, Shan-shan Yang, Jian-wei Gao, Li-ye Zhou, Rui-feng Liang and Cheng-yi Qu

55. Capgras syndrome in Dementia with Lewy Bodies; Papan Thaipisuttikul, Iryna Lobach, Yael Zweig, Ashita Gurnani and James E. Galvin

56. Mibampator (LY451395) randomized clinical trial for agitation/aggression in Alzheimer's disease; Paula T. Trzepacz, Jeffrey Cummings, Thomas Konechnik, Tammy D. Forrester, Curtis Chang, Ellen B. Dennehy, Brian A. Willis, Catherine Shuler, Linda B. Tabas and Constantine Lyketsos

57. Comorbid cardiovascular disease and major depression among ethnic and racial groups in the United States; Hector M. Gonzlez and Wassim Tarraf

58. Development and initial testing of the Person-Centred Health Care for Older Adults Survey; Briony Dow, Marcia Fearn, Betty Haralambous, Jean Tinney, Keith Hill and Stephen Gibson

59. Comorbidity, health status, and quality of life in institutionalized older people with and without dementia; Salom Martn-Garca, Carmen Rodrguez-Blzquez, Iluminada Martnez-Lpez, Pablo Martnez-Martn and Maria Joo Forjaz

60. Risk factors for incident dementia in the very old; Karin Wallin, Gustaf Bostrm, Miia Kivipelto and Yngve Gustafson

61. Patterns of executive dysfunction in amnestic mild cognitive impairment; Nai-Ching Chen, Chiung-Chih Chang, Ker-Neng Lin, Chi-Wei Huang, Wen-Neng Chang, Ya-Ting Chang, Ching Chen, Yen-Chi Yeh and Pei-Ning Wang

62. The Tasmanian Healthy Brain Project (THBP): a prospective longitudinal examination of the effect of university-level education in older adults in preventing age-related cognitive decline and reducing the risk of dementia; Mathew J. Summers, Nichole L.J. Saunders, Michael J. Valenzuela, Jeffery J. Summers, Karen Ritchie, Andrew Robinson and James C. Vickers

63. Quality of life in patients with cognitive impairment: validation of the Quality of LifeAlzheimer's Disease scale in Portugal; Helena Brrios, Ana Verdelho, Sofia Narciso, Manuel Gonalves-Pereira, Rebecca Logsdon and Alexandre de Mendona

64. CERAD practice effects and attrition bias in a dementia prevention trial; Melissa Mathews, Erin Abner, Allison Caban-Holt, Richard Kryscio and Frederick Schmitt

65. Service-related needs of older people with dementia: perspectives of service users and their unpaid carers; Sylwia Grska, Kirsty Forsyth, Linda Irvine, Donald Maciver, Susan Prior, Jacqueline Whitehead, Janice Flockhart, Jane Fairnie and Jenny Reid

66. Two types of squalor: findings from a factor analysis of the Environmental Cleanliness and Clutter Scale (ECCS); John Snowdon, Graeme Halliday and Glenn E. Hunt

67. Cognitive predictors for five-year conversion to dementia in community-dwelling Chinese older adults; Candy H. Y. Wong, Grace T. Y. Leung, Ada W. T. Fung, W. C. Chan and Linda C. W. Lam

68. Role of severity and gender in the association between late-life depression and all-cause mortality; Hyun-Ghang Jeong, Jung Jae Lee, Seok Bum Lee, Joon Hyuk Park, Yoonseok Huh, Ji Won Han, Tae Hui Kim, Ho Jun Chin and Ki Woong Kim

69. Benefits of training working memory in amnestic mild cognitive impairment: specific and transfer effects; Barbara Carretti, Erika Borella, Silvia Fostinelli and Michela Zavagnin

70. Cross-cultural differences in dementia: the Sociocultural Health Belief Model; Philip Sayegh and Bob G. Knight

71. Reconceptualizing models of delirium education: findings of a Grounded Theory study; Andrew Teodorczuk, Elizabeta Mukaetova-Ladinska, Sally Corbett and Mark Welfare

72. Age and synchrony effects in performance on the Rey Auditory Verbal Learning Test; Claire A. Lehmann, Anthony D. G. Marks and Tanya L. Hanstock

73. Effects of education on the progression of early- versus late-stage mild cognitive impairment; Byoung Seok Ye, Sang Won Seo, Hanna Cho, Seong Yoon Kim, Jung-Sun Lee, Eun-Joo Kim, Yunhwan Lee, Joung Hwan Back, Chang Hyung Hong, Seong Hye Choi, Kyung Won Park, Bon D. Ku, So Young Moon, SangYun Kim, Seol-Heui Han, Jae-Hong Lee, Hae-Kwan Cheong and Duk L. Na

74. Fall determinants in older long-term care residents with dementia: a systematic review; Tobias F. Krpelin, Jacques C. L. Neyens, Ruud J. G. Halfens, Gertrudis I. J. M. Kempen and Jan P. H. Hamers

75. Cognitive screening of older adults: the utility of pentagon drawing; Edward Helmes

76. Patterns of dietary intake and psychological distress in older Australians: benefits not just from a Mediterranean diet; Allison Hodge, Osvaldo P. Almeida, Dallas R. English, Graham G. Giles and Leon Flicker

77. Complex visual hallucinations in a Parkinson patient: don't blame James if it's Charles's fault; Kurt Segers

78. Gender differences in health service use for mental health reasons in community dwelling older adults with suicidal ideation; Helen-Maria Vasiliadis, Sarah Gagn, Natalia Jozwiak and Michel Prville

79. Mental health services for black and minority ethnic elders in the United Kingdom: a systematic review of innovative practice with service provision and policy implications; Sarmishtha Bhattacharyya and Susan Mary Benbow

80. Sahlgrenska Academy Self-reported Cognitive Impairment Questionnaire (SASCI-Q) a research tool discriminating between subjectively cognitively impaired patients and healthy controls; Marie Eckerstrm, Johanna Skoogh, Sindre Rolstad, Mattias Gthlin, Gunnar Steineck, Boo Johansson and Anders Wallin

81. Changes in body mass in later life and incident dementia; Brian D. Power, Helman Alfonso, Leon Flicker, Graeme J. Hankey, Bu B. Yeap and Osvaldo P. Almeida

82. Cognitive stimulation therapy (CST): neuropsychological mechanisms of change; Louise Hall, Martin Orrell, Joshua Stott and Aimee Spector

83. The effectiveness of control strategies for dementia-driven wandering, preventing escape attempts: a case report; Daniel Valle Padilla, Mara Teresa Daza Gonzlez, Inmaculada Fernndez Agis, Jenna Strizzi and Raquel Alarcn Rodrguez

84. Communication skills training in dementia care: a systematic review of effectiveness, training content, and didactic methods in different care settings; Eva Eggenberger, Katharina Heimerl and Michael I. Bennett

85. Do changes in coping style explain the effectiveness of interventions for psychological morbidity in family carers of people with dementia? A systematic review and meta-analysis; Ryan Li, Claudia Cooper, Allana Austin and Gill Livingston

86. Dimensions of positive symptoms in late versus early onset psychosis; Oliver Mason, Joshua Stott and Ruth Sweeting

87. Physicians practice and familiarity with treatment for agitation associated with dementia in Israeli nursing homes; Jiska Cohen-Mansfield, Atarah Juravel-Jaffe, Aaron Cohen, Iris Rasooly and Hava Golander

88. Longitudinal relationships between subjective fatigue, cognitive function, and everyday functioning in old age; Feng Lin, Ding-Geng Chen, David E. Vance, Karlene K. Ball and Mark Mapstone

89. Relationship between cortisol level and prevalent/incident cognitive impairment and its moderating factors in older adults; Olivier Potvin, Hlne Forget, Michel Prville, Djamal Berbiche, Yvon C. Chagnon and Carol Hudon

90. National trends (20032009) and factors related to psychotropic medication use in community-dwelling elderly population; P. Carrasco-Garrido, A. Lpez de Andrs, V. Hernndez Barrera, Isabel Jimnez-Trujillo and R. Jimnez-Garca

91. Patterns of neuropsychiatric sub-syndromes in Brazilian and Norwegian patients with dementia; Annibal Truzzi, Ingun Ulstein, Letice Valente, Eliasz Engelhardt, Evandro Silva Freire Coutinho, Jerson Laks and Knut Engedal

92. Pharmacological treatments for neuropsychiatric symptoms of dementia in long-term care: a systematic review; Dallas P. Seitz, Sudeep S. Gill, Nathan Herrmann, Sarah Brisbin, Mark J. Rapoport, Jenna Rines, Kimberley Wilson, Ken Le Clair and David K. Conn

93. Clinical Dementia Rating independently predicted conversion to dementia in a cohort of urban elderly in Brazil; Maria Beatriz Marcondes Macedo Montao, Solange Andreoni and Luiz Roberto Ramos

94. Falls prevention interventions for community-dwelling older persons with cognitive impairment: a systematic review; Heidi Winter, Kerrianne Watt and Nancye May Peel

95. Attitudes of UK psychiatrists to the diagnosis of MCI in clinical practice; Joanne Rodda, Santhosh Dontham Gandhi, Naaheed Mukadam and Zuzana Walker

96. Cross-cultural ageism: ageism and attitudes toward aging among Jews and Arabs in Israel; Yoav S. Bergman, Ehud Bodner and Sara Cohen-Fridel

97. Attitudes of long-term care staff toward dementia and their related factors; Jenny Lee, Elsie Hui, Carolyn Kng and Tung Wai Auyeung

98. Relative preservation of the recognition of positive facial expression happiness in Alzheimer disease; Yohko Maki, Hiroshi Yoshida, Tomoharu Yamaguchi and Haruyasu Yamaguchi

99. Hippocampal and amygdala volumes in an older bipolar disorder sample; Chanaka Wijeratne, Sonal Sachdev, Wei Wen, Olivier Piguet, Darren M. Lipnicki, Gin S. Malhi, Phillip B. Mitchell and Perminder S. Sachdev

100. A meta-analysis of the factor structure of the Geriatric Depression Scale (GDS): the effects of language; Giyeon Kim, Jamie DeCoster, Chao-Hui Huang and Ami N. Bryant

101. The Mini-Cog, Clock Drawing Test, and the Mini-Mental State Examination in a German Memory Clinic: specificity of separation dementia from depression; Monika Milian, Anna-Maria Leiherr, Guido Straten, Stephan Mller, Thomas Leyhe and Gerhard W. Eschweiler

102. Differences between elderly voluntary and involuntary admitted psychiatric patients in Greece; Athanasios Douzenis, Ioannis Michopoulos, Rossetos Gournellis, Christos Christodoulou, Emmanouel N. Rizos, Dionysios Sakkas, Athanasios Karkanias, Ioannis Rontos, Georgios Bouras and Lefteris Lykouras

103. Recruiting older men for geriatric suicide research; Sunil S. Bhar, Shannon Wiltsey-Stirman, David Zembroski, Laura McCray, David W. Oslin, Gregory K. Brown and Aaron T. Beck

104. Benefit of an integrative psychotherapeutic nursing home program to reduce multiple psychiatric symptoms of psychogeriatric patients and caregiver burden after six months of follow-up: a re-analysis of a randomized controlled trial; Ton J. E. M. Bakker, Hugo J. Duivenvoorden, Jacqueline van der Lee, Marcel G. M. Olde Rikkert, Aartjan T. F. Beekman and Miel W. Ribbe

105. AwareCare: a pilot randomized controlled trial of an awareness-based staff training intervention to improve quality of life for residents with severe dementia in long-term care settings; Linda Clare, Rhiannon Whitaker, Robert T Woods, Catherine Quinn, Hannah Jelley, Zoe Hoare, Joan Woods, Murna Downs and Barbara A. Wilson

106. Does posterior cortical atrophy on MRI discriminate between Alzheimer's disease, dementia with Lewy bodies, and normal aging? James O'Donovan, Rosie Watson, Sean J. Colloby, Michael J. Firbank, Emma J. Burton, Robert Barber, Andrew. M. Blamire and John T. O'Brien

107. Gender differences in the relation between depression and social support in later life; C.M. Sonnenberg, D.J.H. Deeg, T.G. van Tilburg, D. Vink, M.L. Stek and A.T.F. Beekman

108. Physical impairments in cognitively impaired older people: implications for risk of falls; Morag E. Taylor, Kim Delbaere, Stephen R. Lord, A. Stefanie Mikolaizak and Jacqueline C. T. Close

109. Interpersonal conflict strategies and their impact on positive symptom remission in persons aged 55 and older with schizophrenia spectrum disorders Journal; Carl I. Cohen, Dishal Solanki and Dimple Sodhi

110. Do cerebral white matter lesions influence the rate of progression from mild cognitive impairment to dementia? Michael E. Devine, J. Andres Saez Fonseca and Zuzana Walker

111. Raised IL-2 and TNF- concentrations are associated with postoperative delirium in patients undergoing coronary-artery bypass graft surgery; Jakub Kazmierski, Andrzej Banys, Joanna Latek, Julius Bourke and Ryszard Jaszewski

112. Emotional reactions toward people with dementia results of a population survey from Germany; Olaf von dem Knesebeck, Matthias C. Angermeyer, Daniel Ldecke and Christopher Kofahl

113. Caregivers' experience of the decision-making process for placing a person with dementia into a nursing home: comparing caregivers from Chinese ethnic minority with those from English-speaking backgrounds; Lauren Caldwell, Lee-Fay Low and Henry Brodaty

114. Dementia, neuropsychiatric symptoms, and the use of psychotropic drugs among older people who receive domiciliary care: a cross-sectional study; Jon N. Wergeland, Geir Selbk, Lisbeth D. Hgset, Ulrika Sderhamn and yvind Kirkevold

115. Variability between nursing homes in prevalence of antipsychotic use in patients with dementia; Bart C. Kleijer, Rob J. van Marum, Dinnus H. M. Frijters, Paul A. F. Jansen, Miel W. Ribbe, Antoine C. G. Egberts and Eibert R. Heerdink

116. Application of the Geriatric Anxiety Inventory-Chinese Version (GAI-CV) to older people in Beijing communities; Yue Yan, Tao Xin, Dahua Wang and Dan Tang

117. World War II-related post-traumatic stress disorder and breast cancer risk among Israeli women: a case-control sudy; Neomi Vin-Raviv, Rachel Dekel, Micha Barchana, Shai Linn and Lital Keinan-Boker

118. Exploring the experiences of people with mild cognitive impairment and their caregivers with particular reference to healthcare a qualitative study; Katherine Dean, Crispin Jenkinson, Gordon Wilcock and Zuzana Walker

119. Staff attitudes towards sexual relationships among institutionalized people with dementia: does an extreme cautionary stance predominate? Feliciano Villar, Montserrat Celdrn, Josep Fab and Rodrigo Serrat

120. Operational definitions of successful aging: a systematic review; Theodore D. Cosco, A. Matthew Prina, Jaime Perales, Blossom C. M. Stephan and Carol Brayne

121. Subjective memory complaints, depressive symptoms and cognition in patients attending a memory outpatient clinic; J. Lehrner, D. Moser, S. Klug, A. Glei, E. Auff, P. Dal-Bianco and G. Pusswald

122. Suicidal ideation in elderly Korean population: a two-year longitudinal study; Hee-Ju Kang, Robert Stewart, Bo-Ok Jeong, Seon-Young Kim, Kyung-Yeol Bae, Sung-Wan Kim, Jae-Min Kim, Il-Seon Shin and Jin-Sang Yoon

123. Wellbeing-enhancing occupation and organizational and environmental contributors in long-term dementia care facilities: an explorative study; Dieneke Smit, Bernadette Willemse, Jacomine de Lange and Anne Margriet Pot

124. Association of cognitive impairment with frailty in community-dwelling older adults; Eun Sook Han, Yunhwan Lee and Jinhee Kim

125. Dysthymic disorder in the elderly population; D. P. Devanand

126. Patients with Korsakoff syndrome in nursing homes: characteristics, comorbidity, and use of psychotropic drugs; Ineke J. Gerridzen and M. Anne Goossensen

127. Stressful life events are not associated with the development of dementia; Anna Sundstrm, Michael Rnnlund, Rolf Adolfsson and Lars-Gran Nilsson

128. Medical students attitudes toward people with dementia: an international investigation; Ellen StClair Tullo and Tony Johnstone Young

129. Factorial validity of the Center for Epidemiologic Studies Depression Scale short form in older population in China; Huajuan Chen and Ada C. Mui

130. A systematic review of metabolic side effects related to the use of antipsychotic drugs in dementia; A.R. Atti, B. Ferrari Gozzi, G. Zuliani, V. Bernabei, P. Scudellari, D. Berardi, D. De Ronchi, I. Tarricone and M. Menchetti

131. Measuring family caregiver efficacy for managing behavioral and psychological symptoms in dementia: a psychometric evaluation; Nadia Crellin, Georgina Charlesworth and Martin Orrell

132. Planning for tomorrow whilst living for today: the views of people with dementia and their families on advance care planning; Claire Dickinson, Claire Bamford, Catherine Exley, Charlotte Emmett, Julian Hughes and Louise Robinson

133. Impact of a structured multidisciplinary intervention on quality of life of older adults with advanced cancer; Megan M. Chock, Maria I. Lapid, Pamela J. Atherton, Simon Kung, Jeff A. Sloan, Jarrett W. Richardson, Matthew M. Clark and Teresa A. Rummans

134. Adjustment, depression, and anxiety in mild cognitive impairment and early dementia: a systematic review of psychological intervention studies; Bridget Regan and Laura Varanelli

135. Consensus and variations in opinions on delirium care: a survey of European delirium specialists; A. Morandi, D. Davis, J. K. Taylor, G. Bellelli, B. Olofsson, S. Kreisel, A. Teodorczuk, B. Kamholz, W. Hasemann, J. Young, M. Agar, S. E. de Rooij, D. Meagher, M. Trabucchi and A. M. MacLullich

136. Clinical outcomes of older depressed patients with and without comorbid neuroticism; David C. Steffens, Douglas R. McQuoid, Moria J. Smoski and Guy G. Potter

137. The stages of driving cessation for people with dementia: needs and challenges; Jacki Liddle, Sally Bennett, Shelley Allen, David C. Lie, Bradene Standen and Nancy A. Pachana

138. More insight into the concept of apathy: a multidisciplinary depression management program has different effects on depressive symptoms and apathy in nursing home; Ruslan Leontjevas, Steven Teerenstra, Martin Smalbrugge, Myrra J.F.J. Vernooij-Dassen, Ernst T. Bohlmeijer, Debby L. Gerritsen and Raymond T.C.M. Koopmans

139. Dementia special care units: a comparison with standard units regarding residents profile and care features; Mara Crespo, Carlos Hornillos and M. Mar Gmez

140. The Stick Design Test on the assessment of older adults with low formal education: evidences of construct, criterion-related and ecological validity; Jonas Jardim de Paula, Mnica Vieira Costa, Matheus Bortolosso Bocardi, Mariana Cortezzi, Edgar Nunes De Moraes and Leandro Fernandes Malloy-Diniz

141. Prevalence of delirium among outpatients with dementia; N. Hasegawa, M. Hashimoto, S. Yuuki, K. Honda, Y. Yatabe, K. Araki and M. Ikeda

142. Program evaluation of a telepsychiatry service for older adults connecting a university-affiliated geriatric center to a rural psychogeriatric outreach service in Northwest Ontario, Canada; David K. Conn, Robert Madan, Jenny Lam, Tim Patterson and Sandy Skirten

143. Incidence of dementia: evidence for an effect modification by gender. The ILSA Study; Marianna Noale, Federica Limongi, Sabina Zambon, Gaetano Crepaldi and Stefania Maggi

144. A literature review of spaced-retrieval interventions: a direct memory intervention for people with dementia; Alexandra S. Creighton, Eva S. van der Ploeg and Daniel W. OConnor

145. Clinical and demographic covariates of chronic opioid and non-opioid analgesic use in rural-dwelling older adults: the MoVIES project; Jordan F. Karp, Ching-Wen Lee, Jonathan McGovern, Gary Stoehr, Chung-Chou H. Chang and Mary Ganguli

146. Substitute consent practices in the face of uncertainty: a survey of Canadian researchers in aging; Gina Bravo, Sheila Wildeman, Marie-France Dubois, Scott YH Kim, Carole Cohen, Janice Graham and Karen Painter

147. Improving the end-of-life for people with dementia living in a care home: an intervention study; Gill Livingston, Elanor Lewis-Holmes, Catherine Pitfield, Monica Manela, Diana Chan, Eleanor Constant, Hannah Jacobs, Gaby Wills, Natasha Carson and Jackie Morris

148. Homocysteine levels and dementia risk in Yoruba and African Americans; Hugh C. Hendrie, Olusegun Baiyewu, Kathleen A. Lane, Christianna Purnell, Sujuan Gao, Ann Hake, Adesola Ogunniyi, Oye Gureje, Frederick W. Unverzagt, Jill Murrell, Mark A. Deeg and Kathleen Hall

149. Determinants of thoughts of death or suicide in depressed older persons; Ista C. H. M. Bogers, Marij Zuidersma, Marjolein L. Boshuisen, Hannie C. Comijs and Richard C. Oude Voshaar

150. The efficacy of a multifactorial memory training in older adults living in residential care settings; Andrea Vrani, Ana Marija pani, Barbara Carretti and Erika Borella

151. Gender differences in the trajectories of late-life depressive symptomology and probable depression in the years prior to death; R. A. Burns, M. A. Luszcz, K. M. Kiely, P. Butterworth, C. Browning, P. Mitchell and K. J. Anstey

152. An insulinoma presenting with hypochondriac delusions and food refusal, Susana Renca, Graa Santos and Joaquim Cerejeira

153. Validity, reliability, and feasibility of the German version of the Caregiver Reaction Assessment scale (G-CRA): a validation study; Astrid Stephan, Herbert Mayer, Anna Renom Guiteras and Gabriele Meyer

154. Dyadic interventions for community-dwelling people with dementia and their family caregivers: a systematic review; Netta Van't Leven, Anna-Eva J. C. Prick, Johanna G. Groenewoud, Pepijn D. D. M. Roelofs, Jacomine de Lange and Anne Margriet Pot

155. The association of A amyloid and composite cognitive measures in healthy older adults and MCI; Karra D. Harrington, Yen Ying Lim, Kathryn A. Ellis, Carly Copolov, David Darby, Michael Weinborn, David Ames, Ralph N. Martins, Greg Savage, Cassandra Szoeke, Christopher Rowe, Victor L. Villemagne, Colin L. Masters and Paul Maruff

156. Assessing quality of life of nursing home residents with dementia: feasibility and limitations in patients with severe cognitive impairment; Mara Crespo, Carlos Hornillos and M. Mar Gmez

157. Prevalence of insomnia and associated factors in a community sample of elderly individuals in South Korea; Won-Hyoung Kim, Byung-Soo Kim, Shyn-Kyum Kim, Sung-Man Chang, Dong-Woo Lee, Maeng-Je Cho,Jae-Nam Bae

158. Predictors of agreement between general practitioner detection of dementia and the revised Cambridge Cognitive Assessment (CAMCOG-R); C. Dimity Pond, Karen E. Mate, Jill Phillips, Nigel P. Stocks, Parker J. Magin, Natasha Weaver and Henry Brodaty

159. Cohort differences in dementia recognition and treatment indicators among assisted living residents in Maryland: did a change in the resident assessment tool make a difference? Quincy M. Samus, Amrita Vavilikolanu, Lawrence Mayer, Matthew McNabney, Jason Brandt, Constantine G. Lyketsos and Adam Rosenblatt

160. Social networking sites and older users a systematic review; Tobias Nef, Raluca L. Ganea, Ren M. Mri and Urs P. Mosimann

161. Aspects of awareness in patients with Alzheimers disease; Selina Mrdh, Thomas Karlsson and Jan Marcusson

162. A psychological pathway from insomnia to depression among older adults; Paul Sadler, Suzanne McLaren and Megan Jenkins

163. The Brazilian version of the Neuropsychiatric Inventory-Clinician rating scale (NPI-C): reliability and validity in dementia; Florindo Stella, Orestes Vicente Forlenza, Jerson Laks, Larissa Pires de Andrade, Michelle A. Ljubetic Avendao, Elisandra Villela Gasparetto S, Joo de Castilho Cao, Constantine G. Lyketsos and Kate de Medeiros

164. Familiarity, knowledge, and preferences of family physicians regarding mild cognitive impairment; Perla Werner, Jeremia Heinik and Eliezer Kitai

165. A case in which mirtazapine reduced auditory hallucinations in a patient with Parkinson disease; Tomoyuki Nagata, Shunichiro Shinagawa, Kenji Tagai and Kazuhiko Nakayama

166. A randomized crossover trial to study the effect of personalized, one-to-one interaction using Montessori-based activities on agitation, affect, and engagement in nursing home residents with Dementia; Eva S. van der Ploeg, Barbara Eppingstall, Cameron J. Camp, Susannah J. Runci, John Taffe and Daniel W. O'Connor

167. Reliability and validity of the Attitudes to Ageing Questionnaire (AAQ) in older people in Spain; Ramona Lucas-Carrasco, Ken Laidlaw, Juana Gmez-Benito and Michael J. Power

168. COST: Cognitive State Test, a brief screening battery for Alzheimer disease in illiterate and literate patients; Gulsen Babacan-Yildiz, Ahmet T. Isik, Emel Ur, Emine Aydemir, Can Ertas, Merve Cebi, Pinar Soysal, Esra Gursoy, Mehmet Kolukisa, Gulsen Kocaman and Arif Celebi

169. Self-reported depressive syndromes in mild cognitive impairment and mild Alzheimer's disease; Janessa O. Carvalho, Jing Ee Tan, Beth A. Springate and Jennifer D. Davis

170. The Birmingham Relationship Continuity Measure: the development and evaluation of a measure of the perceived continuity of spousal relationships in dementia; Gerard A. Riley, Gemma Fisher, Barbara F. Hagger, Amy Elliott, Hannah Le Serve and Jan R. Oyebode

171. Utility of neuropsychiatric tools in the differential diagnosis of dementia with Lewy bodies and Alzheimer's disease: quantitative and qualitative findings; Aida Surez-Gonzlez, Alberto Serrano-Pozo, Eva M. Arroyo-Anll, Emilio Franco-Macas, Juan Polo, David Garca-Sols and Eulogio Gil-Nciga

172. Acceptance of and attitudes towards Alzheimer's disease screening in elderly German adults; Sarah R. Braun, Katinka Reiner, Christina Tegeler, Nina Bucholtz, Malaz A. Boustani and Elisabeth Steinhagen-Thiessen

173. The development and validation of a patient-reported quality of life measure for people with mild cognitive impairment; Katherine Dean, Crispin Jenkinson, Gordon Wilcock and Zuzana Walker

174. Measuring anxiety about aging across the adult lifespan; Kerry A. Sargent-Cox, Masori Rippon and Richard A. Burns

175. Validation of the Caregiver Guilt Questionnaire (CGQ) in a sample of British dementia caregivers; Louise Roach, Ken Laidlaw, David Gillanders and Kathryn Quinn

176. Neuroimaging of depression in Parkinson's disease: a review; Marcos Hortes N. Chagas, Ila M.P. Linares, Giovana Jorge Garcia, Jaime E.C. Hallak, Vitor Tumas and Jos Alexandre S. Crippa

177. Comparison of the Montreal Cognitive Assessment and the Mini-Mental State Examination in detecting multi-domain mild cognitive impairment in a Chinese sub-sample drawn from a population-based study; YanHong Dong, Wah Yean Lee, Saima Hilal, Monica Saini, Tien Yin Wong, Christopher Li-Hsian Chen, Narayanaswamy Venketasubramanian and Mohammad Kamran Ikram

178. The significance of experiences of war and migration in older age: long-term consequences in child survivors from the Dutch East Indies; Trudy T. M. Mooren and Rolf J. Kleber

179. The development and evaluation of the DK-20: a knowledge of dementia measure; Niamh Shanahan, Martin Orrell, Astrid K Schepers and Aimee Spector

180. Validation of the General Practitioner Assessment of Cognition Chinese version (GPCOG-C) in China; Xia Li, Shifu Xiao, Yuan Fang, Minjie Zhu, Tao Wang, Katrin Seeher and Henry Brodaty

181. Caregiver personality predicts rate of cognitive decline in a community sample of persons with Alzheimers disease. The Cache County Dementia Progression Study; Maria C. Norton, Christine Clark, Elizabeth B. Fauth,Kathleen W. Piercy, Roxane Pfister, Robert C. Green, Christopher D. Corcoran, Peter V. Rabins, Constantine G. Lyketsos and JoAnn T. Tschanz

182. Aberrant topographical organization in gray matter structural network in late life depression: a graph theoretical analysis; Hyun Kook Lim, Won Sang Jung and Howard J Aizenstein

Journal of Attention Disorders

1. Neuropsychological Functioning in Adults With ADHD and Adults With Other Psychiatric Disorders: The Issue of Specificity; Ylva Holst and Lisa B. Thorell

2. Adult ADHD Among NSW Prisoners: Prevalence and Psychiatric Comorbidity; Elizabeth Moore, Sandra Sunjic, Sharlene Kaye, Vicki Archer and Devon Indig

3. Attention Problems and Academic Achievement: Do Persistent and Earlier-Emerging Problems Have More Adverse Long-Term Effects? David L. Rabiner, Madeline M. Carrig, and Kenneth A. Dodge

4. The Impact of Coexisting Emotional and Conduct Problems on Family Functioning and Quality of Life Among Adolescents With ADHD; Jorun Schei, Thomas Jozefiak, Torunn Stene Novik, Stian Lydersen and Marit S. Indredavik

5. Variation in Latent Classes of Adult AttentionDeficit Hyperactivity Disorder by Sex and Environmental Adversity; Jane L. Ebejer, Sarah E. Medland, Julius van der Werf, Michael Lynskey, Nicholas G. Martin and David L. Duffy

6. Empirically Determined, Psychopathological Subtypes in Children With ADHD; Yvonne Zenglein, Christina Schwenck, Eva Westerwald, Catharina Schmidt, Sonja Beuth, Jobst Meyer, Haukur Palmason, Christiane Seitz, Susann Hnig and Christine M. Freitag

7. Reading Performance of Young Adults With ADHD Diagnosed in Childhood: Relations With Executive Functioning; Ana Miranda, Jessica Mercader, M. Inmaculada Fernndez and Carla Colomer

8. Attenuated Readiness Potential in the Absence of Executive Dysfunction in Adults With ADHD; Bo-Kyung Seo, Gudrun Sartory, Bernhard Kis, Norbert Scherbaum and Bernhard W. Mller

9. Training Executive, Attention, and Motor Skills: A Proof-of-Concept Study in Preschool Children With ADHD; Jeffrey M. Halperin, David J. Marks, Anne-Claude V. Bedard, Anil Chacko, Jocelyn T. Curchack, Carol A. Yoon and Dione M. Healey

10. German Validation of the Conners Adult ADHD Rating ScaleSelf-Report: Confirmation of Factor Structure in a Large Sample of Participants With ADHD; Hanna Christiansen, Oliver Hirsch, Alexandra Philipsen, Robert D. Oades, Swantje Matthies, Johannes Hebebrand, Jennifer Ueckermann, Mona Abdel-Hamid, Markus Kraemer, Jens Wiltfang, Erika Graf, Michael Colla, Esther Sobanski, Barbara Alm, Michael Rsler, Christian Jacob, Thomas Jans, Michael Huss, Benno G. Schimmelmann and Bernhard Kis

11. Adolescent Outcome of Child ADHD in Primary Care Setting: Stability of Diagnosis; Tomasz Srebnicki, Artur Koakowski, and Tomasz Wolan czyk

12. Atomoxetine for Treating ADHD Symptoms in Autism: A Systematic Review; Ahmad Ghanizadeh

13. Prediction of ADHD to Anxiety Disorders: An 11-Year National; Insurance Data Analysis in Taiwan, Yueh-Ming Tai, Churn-Shiouh Gau, Susan Shur-Fen Gau and Hung-Wen Chiu

14. Toward Defining the Neural Substrates of ADHD: A Controlled Structural MRI Study in Medication-Nave Adults; Nikos Makris, Lichen Liang, Joseph Biederman, Eve M. Valera, Ariel B. Brown, Carter Petty, Thomas J. Spencer, Stephen V. Faraone, and Larry J. Seidman

15. Cognitive Processes in ADHD and Aspergers Disorder: Overlaps and Differences in PASS Profiles; Stefano Taddei and Bastianina Contena

16. Efficacy and Safety of Atomoxetine Hydrochloride in Asian Adults With ADHD: A Multinational 10-Week Randomized Double-Blind Placebo-Controlled Asian Study; Taro Goto, Yuko Hirata, Yasushi Takita, Paula T. Trzepacz, Albert J. Allen, Dong-Ho Song, Susan Shur-Fen Gau, Hironobu Ichikawa and Michihiro Takahashi

17. Are There Executive Dysfunction Subtypes Within ADHD? Bethan A. Roberts, Michelle M. Martel and Joel T. Nigg

18. Long-Term Memory Performance in Adult ADHD: A Meta-Analysis; Timo Skodzik, Heinz Holling and Anya Pedersen

19. Do 5-Year-Old Children Perform Dual-Task Coordination Better Than AD Patients? Maria Victoria Sebastian and Laura Hernndez-Gil

20. Oxidative Stress and ADHD: A Meta-Analysis; Nidhin Joseph, Yanli Zhang-James, Andras Perl and Stephen V. Faraone

21. Behavioral and Emotional Problems Associated With Convergence Insufficiency in Children: An Open Trial; Eric Borsting, G. Lynn Mitchel, L. Eugene Arnold, Mitchell Scheiman, Christopher Chase, Marjean Kulp, Susan Cotter and CITT-RS Group

22. Does Gender Moderate the Relations Between Externalizing Behavior and Key Emergent Literacy Abilities? Evidence From a Longitudinal Study; Nicholas P. Allan, Shauna W. Joye and Christopher J. Lonigan

23. The Association Between ADHD and Antisocial Personality Disorder (ASPD): A Review; Ole Jakob Storeb and Erik Simonsen

24. Maternal Ratings of the ADHD Symptoms: Subtypes Versus Severity in Clinic-Referred Children and Adolescents; Rapson Gomez, Alasdair Vance and Rashika Miranjani Gomez

25. A Pilot Trial of Mindfulness Meditation Training for ADHD in Adulthood: Impact on Core Symptoms, Executive Functioning, and Emotion Dysregulation; John T. Mitchell, Elizabeth M. McIntyre, Joseph S. English, Michelle F. Dennis, Jean C. Beckham and Scott H. Kollins

26. Are Classrooms Meeting the Basic Psychological Needs of Children With ADHD Symptoms? A Self-Determination Theory Perspective; Maria Rogers and Rosemary Tannock

27. The Prevalence of ADHD in a Population-Based Sample; Andrew S. Rowland, Betty J. Skipper, David M. Umbach, David L. Rabiner, Richard A. Campbell, Albert J. Naftel and Dale P. Sandler

28. Stigmatization of ADHD: A Developmental Review; Matthew S. Lebowitz

29. ADHD Symptoms Across the Lifespan: A Comparison of Symptoms Captured by the Wender and Barkley Scales and DSM-IV Criteria in a Population-Based Swedish Sample Aged 65 to 80; Taina Guldberg-Kjr and Boo Johansson

30. Diagnostic and Demographic Differences Between Incarcerated and Nonincarcerated Youth (Ages 6-15) With ADHD in South Carolina; Samuel L. Soltis, Janice Probst, Sudha Xirasagar, Amy B. Martin and Bradley H. Smith

31. Postsecondary ADHD Documentation Requirements: Common Practices in the Context of Clinical Issues, Legal Standards, and Empirical Findings; Will Lindstrom, Jason M. Nelson and Patricia Foels

32. Neurobiology of ADHD From Childhood to Adulthood: Findings of Imaging Methods; Tomas Kasparek, Pavel Theiner and Alena Filova

33. ADHD Preschoolers With and Without ODD: Do They Act Differently Depending On Degree of Task Engagement/Reward? Chaya B. Gopin, Olga Berwid, David J. Marks, Agnieska Mlodnicka and Jeffrey M. Halperin

34. A Controlled Trial of a Cognitive Skills Program for Personality-Disordered Offenders; Susan Young, Gareth Hopkin, Derek Perkin, Catherine Farr, Amie Doidge and Gisli Gudjonsson

35. Change Blindness in Children With ADHD: A Selective Impairment in Visual Search? Lisa Maccari, Maria Casagrande, Diana Martella, Mariagrazia Anolfo, Caterina Rosa, Luis J. Fuentes and Augusto Pasini

36. A Retrospective Survey of Childhood ADHD Symptomatology Among Adult Narcoleptics; Edward J. Modestino and Jeanna Wincheste

37. Future Research Directions in Sleep and ADHD: Report of aConsensus Working Group Judith Owens, Reut Gruber, Thomas Brown, Penny Corkum, Samuele Cortese, Louise OBrien, Mark Stein and Margaret Weiss

38. Case Reports of Sleep Phenotypes of ADHD: From Hypothesis to Clinical Practice Silvia Miano, Renato Donfrancesco, Pasquale Parisi, Jole Rabasco, Anna Rita Mazzotta, Alessandra Tabarrini, Ottavio Vitelli and Maria Pia Villa

39. Meta-Analysis: Parental Interventions for Preschool ADHD; Jilian M. Mulqueen, Christine A. Bartley and Michael H. Bloch

40. Morning and Evening Effects of Guanfacine Extended Release Adjunctive to Psychostimulants in Pediatric ADHD: Results From a Phase III Multicenter Trial; Timothy E. Wilens, Keith McBurnett, John Turnbow, Thomas Rugino, Carla White and Sharon Youcha

41. Association Between Insecure Attachment and ADHD: Environmental Mediating Factors; Ole Jakob Storeb, Pernille Darling Rasmussen and Erik Simonsen

42. A Review of Peer Relationships and Friendships in Youth With ADHD; Denise M. Gardner and Alyson C. Gerdes

43. Daily Symptom Profiles of Children With ADHD Treated With Modified-Release Methylphenidate: An Observational Study; Christopher Hautmann, Aribert Rothenberger and Manfred Dpfner

44. Parents Perspectives About Factors Influencing Adherence to Pharmacotherapy for ADHD; Rana Ahmed, Jacqueline Borst, Yong C. Wei and Parisa Aslani

45. Lobeline Effects on Cognitive Performance in Adult ADHD; Catherine A. Martin, Paul A. Nuzzo, John D. Ranseen, Mark S. Kleven, Greg Guenthner, Yolanda Williams, Sharon L. Walsh and Linda P. Dwoskin

46. Effect of External Irrelevant Distracters on a Visual Search Test in School-Age Children: Computerized Assessment; M. A. Quiroga, J. Santacreu, C. Lpez-Cavada, E. Capote and D. Morillo

47. Language Delay in 3-Year-Old Children With ADHD Symptoms; Nina Rohrer-Baumgartner, Pl Zeiner, Patricia Eadie, Jens Egeland, Kristin Gustavson, Ted Reichborn-Kjennerud and Heidi Aase

48. Prevalence and Smoking Behavior Characteristics of Nonselected Smokers With Childhood and/or Adult Self-Reported ADHD Symptoms in a Smoking-Cessation Program: A Cross-Sectional Study; Guillaume Fond, Sebastien Guillaume, Isabelle Jaussent, Severine Beziat, Alexandra Macgregor, Paquito Bernard, Philippe Courtet, Daniel Bailly and Xavier Quantin

49. Comparative Effects of Emotion Management Training and Social Skills Training in Korean Children With ADHD; Eun Sil Choi and Woo Kyeong Lee

50. A Neuropsychological Perspective on Attention Problems in Neurofibromatosis Type 1; Alexandra K.Templer, Jeffrey B. Titus and David H. Gutmann

51. ADHD Medication Vacations and Parent Child Interactions by Gender; Lucy Barnard-Brak, Marcelo Schmidt and Tracey Sulak

52. Nonmedical Use of Prescription ADHD Stimulant Medications Among Adults in a Substance Abuse Treatment Population: Early Findings From the NAVIPPRO Surveillance System; Theresa A. Cassidy, Emily C. McNaughton, Sajan Varughese, Leo Russo, Mirella Zulueta and Stephen F. Butler

53. Physical Activity, Affect, and Cognition in Children With Symptoms of ADHD; Caterina Gawrilow, Gertraud Stadler, Nadine Langguth, Alexander Naumann and Antje Boeck

54. Assessment of ADHD Symptoms and the Issue of Cultural Variation: Are Conners 3 Rating Scales Applicable to Children and Parents With Migration Background? Martin Schmidt, Verena Reh, Oliver Hirsch, Winfried Rief and Hanna Christiansen

55. Attributions and Perception of Methylphenidate Effects in Adolescents With ADHD; William E. Pelham Jr., Elizabeth M. Gnagy, Margaret H. Sibley, Heidi L. Kipp, Bradley H. Smith, Steven W. Evans and Oscar Bukstein

56. ADHD Diagnosis: As Simple As Administering a Questionnaire or a Complex Diagnostic Process? Ashton Parker and Penny Corkum

57. Overestimation of Physical Abilities Among Boys With and Without ADHD Sarah A. Helseth, Beth S. Bruce, and Daniel A. Waschbusch

58. The Effects of Preresponse Cues on Inhibitory Control and Response Time in Adults With ADHD; Walter Roberts, Richard Milich and Mark T. Fillmore

59. A Multicenter, Open-Label Trial to Evaluate the Quality of Life in Adults With ADHD Treated With Long-Acting Methylphenidate (OROS MPH): Concerta Quality of Life (CON-QoL) Study; Paulo Mattos, Mrio Rodrigues Louz, Andr Lus Fernandes Palmini, Irismar Reis de Oliveira and Fbio Lopes Rocha

60. A Proposed Multisite Double-Blind Randomized Clinical Trial of Neurofeedback for ADHD: Need, Rationale, and Strategy The Collaborative Neurofeedback Group

61. Introduction: EEG Brain Waves: A Wave of the Future or Past?; no author; Special Section on EEG and ADHD

62. EEG Neurofeedback for ADHD: Double- Blind Sham-Controlled Randomized Pilot Feasibility Trial; L. Eugene Arnold, Nicholas Lofthouse, Sarah Hersch, Xueliang Pan,Elizabeth Hurt, Bethany Bates, Kathleen Kassouf, Stacey Moone and Cara Grantier

63. Diminished Infant P50 Sensory Gating Predicts Increased 40-Month-Old Attention, Anxiety/Depression, and Externalizing Symptoms; Amanda K. Hutchison, Sharon K. Hunter, Brandie D. Wagner, Elizabeth A. Calvin, Gary O. Zerbe and Randal G. Ross

64. Construct Validity and Diagnostic Utility of the Cognitive Assessment System for ADHD; Gary L. Canivez and Allison R. Gaboury

65. Factorial Validity of the ADHD Adult Symptom Rating Scale in a French Community Sample: Results From the ChiP-ARD Study; Alexandre J. S. Morin, Antoine Tran and Herv Caci

66. Mindfulness Training as an Adjunct to Evidence-Based Treatment for ADHD Within Families; Andrew R. Cassone

67. Long-Term Outcomes of ADHD: A Systematic Review of Self-Esteem and Social Function; V. Harpin, L. Mazzone, J. P. Raynaud, J. Kahle and P. Hodgkins

68. The Association Between Hospital-Treated Injuries and ADHD Symptoms in Childhood and Adolescence: A Follow-Up Study in the Northern Finland Birth Cohort 1986; Tuula Hurtig, Hanna Ebeling, Jari Jokelainen, Heli Koivumaa-Honkanen and Anja Taanila

69. Exercise: Applications to Childhood ADHD; Sharon B. Wigal, Natasha Emmerson, Jean-G. Gehricke and Pietro Galassetti

70. WISC-IV Profiles Are Associated With Differences in Symptomatology and Outcome in Children With ADHD; Nicholas S. Thaler, Danielle T. Bello and Lewis M. Etcoff

71. Psychostimulant and Sensory Stimulation Interventions That Target the Reading and Math Deficits of Students With ADHD Sydney S. Zentall, Kinsey Tom-Wright and Jiyeon Lee

72. Lack of Association of Handedness With Inattention and Hyperactivity Symptoms in ADHD; Ahmad Ghanizadeh

73. ADHD and Hyperkinetic Disorder Symptoms in Australian Adults: Descriptive Scores, Incidence Rates, Factor Structure, and Gender Invariance; Rapson Gomez

74. Assessing Attention Deficit by Binocular Rivalry; Juan Antonio Amador-Campos, J. Antonio Aznar-Casanova, Juan Jairo Ortiz-Guerra, Manuel Moreno-Snchez and Antonio Medina-Pea

75. Effects of an 8-Session Behavioral Parent Training Group for Parents of Children With ADHD on Child Impairment and Parenting Confidence; Richard E. A. Loren, Aaron J. Vaughn, Joshua M. Langberg, Jessica E. M. Cyran, Tara Proano-Raps, Beverly H. Smolyansky, Leanne Tamm and Jeffery N. Epstein

76. Effects of Extended Time for College Students With and Without ADHD; Laura A. Miller, Lawrence J. Lewandowski and Kevin M. Antshel

77. The Impact of Idle Time in the Classroom: Differential Effects on Children With ADHD; Lindita Imeraj, Inge Antrop, Herbert Roeyers, Dirk Deboutte, Ellen Deschepper, Sarah Bal and Edmund Sonuga-Barke

78. Validation of the AQT ColorForm Additive Model for Screening and Monitoring Pharmacological Treatment of ADHD; Niels Peter Nielsen and Elisabeth Hemmersam Wiig

79. Self-Control in Postsecondary Settings: Students Perceptions of ADHD College Coaching; David R. Parker, Sharon Field Hoffman, Shlomo Sawilowsky and Laura Rolands

80. Effectiveness of a Program Using a Vehicle Tracking System, Incentives, and Disincentives to Reduce the Speeding Behavior of Drivers With ADHD; Paula T. Markham , Bryan E. Porter, and J. D. Bal

81. Validity of the ADHD Bifactor Model in General Community Samples of Adolescents and Adults, and a Clinic-Referred Sample of Children and Adolescents; Rapson Gomez, Alasdair Vance and Rashika Miranjani Gomez

82. Case Series: Evaluation of Behavioral Sleep Intervention for Medicated Children With ADHD; Jayachandran Vetrayan, Suhana Othman and Smily Jesu Priya Victor Paulraj

83. Obstructive Sleep Apnea Mimics Attention Deficit Disorder; Lauri Blesch and Sarah J. Breese McCoy

84. Relationship Between Sleep Problems and Quality of Life in Children With ADHD; Esra Yrmez and Birim Gnay Kl

85. Sleep and Circadian Rhythmicity in Adult ADHD and the Effect of Stimulants: A Review of the Current Literature; Mark A. Snitselaar , Marcel G. Smits, Kristiaan B. van der Heijden and Jan Spijker

86. Malnutrition and Obesity in Children With ADHD; Serdal Gngr, zg Suna Celiloglu, Sabiha Gngr Raif, zlem zel zcan and Mukadder Ayse Selimog lu

87. The Effectiveness of Short- and Long-Acting Stimulant Medications for Adolescents With ADHD in a Naturalistic Secondary School Setting; William E. Pelham, Bradley H. Smith, Steven W. Evans, Oscar Bukstein, Elizabeth M. Gnagy, Andrew R. Greiner and Margaret H. Sibley

88. Teacher Ratings of the ADHD-RS IV in a Community Sample: Results From the ChiP-ARD Study; Herv M. Caci, Alexandre J. Morin and Antoine Tran

89. Toward Quality Care in ADHD: Defining the Goals of Treatment; Anthony Rostain, Peter S. Jensen, Daniel F. Connor, Laura M. Miesle and Stephen V. Faraone

90. Methylphenidate Reduces State Anxiety During a Continuous Performance Test That Distinguishes Adult ADHD Patients From Controls; Yuval Bloch, Shai Aviram, Aviv Segev, Uri Nitzan, Yechiel Levkovitz, Yoram Braw and Aviva Mimouni Bloch

91. ADHD Symptom Rebound and Emotional Lability With Lisdexamfetamine Dimesylate in Children Aged 6 to 12 Years; Frank A. Lpez, Ann Childress, Ben Adeyi, Bryan Dirks, Thomas Babcock, Brian Scheckner, Robert Lasser, John Shepski and Valerie Arnold

92. Agreement Among Categorical, Dimensional, and Impairment Criteria for ADHD and Common Comorbidities; Joyce Sprafkin, Elizabeth Steinberg, Kenneth D. Gadow and Deborah A.G. Drabick

93. Comorbidity of Allergic and Autoimmune Diseases Among Patients With ADHD: A Nationwide Population-Based Study; Mu-Hong Chen, Tung-Ping Su, Ying-Sheue Chen, Ju-Wei Hsu, Kai-Lin Huang,Wen-Han Chang, Tzeng-Ji Chen and Ya-Mei Bai

94. Comparison of On-Road Driving Between Young Adults With and Without ADHD; Richard L. Merkel, Jr., J. Quyen Nichols, Jonathan C. Fellers, Priscilla Hidalgo, Lady A. Martinez, Ivan Putziger, Roger C. Burket and Daniel J. Cox

95. Attentional Networks in Boys With ADHD or Autism Spectrum Disorder and the Relationship With Effortful Control ; Vicky Samyn, Herbert Roeyers, Patricia Bijttebier and Jan R. Wiersema

96. Behavioral Assessment of Core ADHD Symptoms Using the QbTest; Verena Reh,Martin Schmidt, Le Lam, Benno G. Schimmelmann, Johannes Hebebrand, Winfried Rief, and Hanna Christiansen

97. Is ADHD a Risk Factor for High School Dropout? A Controlled Study; Ronna Fried, Carter Petty, Stephen V. Faraone, Laran L. Hyder, Helen Day, and Joseph Biederman logistic regression

98. Assessing ADHD in Latino Families: Evidence for Moving Beyond Symptomatology; Alyson C. Gerdes, Kathryn E. Lawton, Lauren M. Haack,

99. Are Some Individuals Diagnosed With ADHD Prone to Alcohol Abuse?: Consideration of Two Possible Mediating Factors for This Susceptibility; Allison Maxwell

100. Exasperating or Exceptional? Parents Interpretations of Their Childs ADHD Behavior; Heather C. Lench, Linda J. Levine and Carol K. Whalen

101. The Childhood Executive Function Inventory: Confirmatory Factor Analyses and Cross-Cultural Clinical Validity in a Sample of 8- to 11-Year-Old Childre; Corinne Catale, Thierry Meulemans and Lisa B. Thorell

102. ADHD Undetected in Criminal Adults; Nannet J. L. Buitelaar and Robert F. Ferdinand

103. Behavior Rating Inventory of Executive FunctioningPreschool (BRIEF-P) Applied to Teachers: Psychometric Properties and Usefulness for Disruptive Disorders in 3-Year-Old Preschoolers; Lourdes Ezpeleta, Roser Granero, Eva Penelo, Nria de la Osa, and Josep M. Domnech

104. Could Polyunsaturated Fatty Acids Deficiency Explain Some Dysfunctions Found in ADHD? Hypotheses From Animal Research; Catherine Transler, Siobhan Mitchell and Ans Eilander

105. Factor Analysis of Five Adult ADHD Self-Report Measures: Are They All the Same? P. Dennis Rodriguez and Stephanie L. Simon-Dack

106. Pilot Physical Activity Intervention Reduces Severity of ADHD Symptoms in Young Children; Alan L. Smith, Betsy Hoza, Kate Linnea, Julia D. McQuade, Meghan Tomb, Aaron J. Vaughn, Erin K. Shoulberg, and Holly Hook

107. DSM-IV ADHD Symptoms Self- Ratings by Adolescents: Test of Invariance Across Gender; Rapson Gomez

108. Ecological and Highly Demanding Executive Tasks Detect Real-Life Deficits in High-Functioning Adult ADHD Patients; Teresa Torralva, Ezequiel Gleichgerrcht, Alicia Lischinsky, Mara Roca and Facundo Manes

109. Psychopathology and Personality in Parents of Children With ADHD; Hans-Christoph Steinhausen, Julia Gllner, Daniel Brandeis, Ueli C. Mller, Lilian Valko and Renate Drechsler

110. A Novel Group Therapy for Children With ADHD and Severe Mood Dysregulation; Gregory A. Fabiano, Opeolowa O. Akinnusi, Jenifer L. Haak and William E. Pelham James G. Waxmonsky, Fran A. Wymbs, Meaghan E. Pariseau, Peter J. Belin, Daniel A. Waschbusch, Lysett Babocsai

111. Efficacy of Atomoxetine for the Treatment of ADHD Symptoms in Patients With Pervasive Developmental Disorders: A Prospective, Open-Label Study Alberto Fernndez-Jan, Daniel Martn Fernndez-Mayoralas, Beatriz Calleja-Prez, Nuria Muoz-Jareo, Mara del Rosario Campos Daz, and Sonia Lpez-Arribas

112. Comparison of Mother, Father, and Teacher Reports of ADHD Core Symptoms in a Sample of Child Psychiatric Outpatients; Henrik Sollie, Bo Larsson, and Willy-Tore Mrch

113. Brain Cortical Thickness in ADHD: Age, Sex and Clinical Correlations, Luis Guillermo Almeida Montes, Hugo Prado Alcntara, Reyna Beatriz Martnez Garca, Lzaro Barajas De La Torre, David vila Acosta and Martn Gallegos Duarte

114. ADHD Symptoms and Benefit From Extended Time Testing Accommodations; Benjamin J. Lovett and Ashley M. Leja

115. Dissociable Response Inhibition in Children With Tourettes Syndrome Compared With Children With ADHD; Kjell Tore Hovik, Kerstin J. Plessen, Erik Winther Skogli, Per Normann, Andersen and Merete ie

116. Response Inhibition in Tic Disorders: Waiting to Respond Is Harder When ADHD Is Present; Marc Thibeault, Martin Lemay, Sylvain Chouinard, Paul Lesprance, Guy A. Rouleau and Francois Richer

117. Effects of Biological Versus Psychosocial Explanations on Stigmatization of Children With ADHD; Matthew S. Lebowitz, Jessica E. Rosenthal, and Woo-kyoung Ahn

118. Factor Structure of Attention Capacities Measured With Eye-Tracking Tasks in 18-Month-Old Toddlers; Marjanneke de Jong, Marjolein Verhoeven, Ignace T. C. Hooge and Anneloes L. van Baar

119. Communicative Perspective-Taking Performance of Adults With ADHD Symptoms; Elizabeth S. Nilsen, Tracy Anne Mewhort Buist, Randall Gillis and Jonathan Fugelsang

120. Canine-Assisted Therapy for Children With ADHD: Preliminary Findings From The Positive Assertive Cooperative Kids Study; Sabrina E. B. Schuck, Natasha A. Emmerson, Aubrey H. Fine and Kimberley D. Lakes

121. Development of a Very Brief Measure of ADHD: The CHAOS Scale; Jess D. Levy, William G. Kronenberger and David W. Dunn

122. Ego Defense Mechanisms and Types of Object Relations in Adults With ADHD; Vanessa de Almeida Silva, Mario Rodrigues Louz, Maria Aparecida da Silva and Eduardo Yoshio Nakano

123. The RAPID Cognitive-Behavioral Therapy Program for Inattentive Children: Preliminary Findings; Susan Young

124. Vulnerability to the Irrelevant Sound Effect in Adult ADHD; Marie-France Pelletier, Helen M. Hodgetts, Martin F. Lafleur, Annick Vincent and Sbastien Tremblay

125. ADHD Knowledge, Misconceptions, and Treatment Acceptability; Mark J. Sciutto

126. Significance of Dopaminergic Gene Variants in the Male Biasness of ADHD; Aneek Das Bhowmik, Kanyakumarika Sarkar, Paramita Ghosh, Manali Das, Nipa Bhaduri, Keka Sarkar, Anirban Ray, Swagata Sinha and Kanchan Mukhopadhyay

127. Characterization of the Theta to Beta Ratio in ADHD: Identifying Potential Sources of Heterogeneity; Sandra K. Loo, Alexander Cho, T. Sigi Hale, James McGough,James McCracken and Susan L. Smalley

128. Concordance Among Physical Educators, Teachers, and Parents Perceptions of Attention Problems in Children; Maria Efstratopoulou, Johan Simons and Rianne Janssen

129. Can the Error-Monitoring System Differentiate ADHD From ADHD With Reading Disability? Reading and Executive Dysfunction as Reflected in Error Monitoring; Tzipi Horowitz-Kraus

130. Effectiveness and Duration of Effect of Open-Label Lisdexamfetamine Dimesylate in Adults With ADHD; Lenard A. Adler, Lauren R. Lynch, David M. Shaw, Samantha P. Wallace, Katherine E. ODonnell, Michael A. Ciranni, Alexis M. Briggie and Stephen V. Faraone

131. Iron and ADHD: Time to Move Beyond Serum Ferritin Levels; Renato Donfrancesco, Pasquale Parisi, Nicola Vanacore, Francesca Martines, Vittorio Sargentini and Samuele Cortese

132. College Students With ADHD Traits and Their Language Styles; Kyungil Kim, Seongjik Lee and Changhwan Lee

133. Assessing Children at Risk: Psychometric Properties of the Motor Behavior Checklist Maria Efstratopoulou, Rianne Janssen and Johan Simons

134. Social Interaction Behavior in ADHD in Adults in a Virtual Trust Game; Stefanie Lis, Nina Baer, Nele Franzen, Meike Hagenhoff, Maika Gerlach, Georgia Koppe, Gebhard Sammer, Bernd Gallhofer and Peter Kirsch

135. ADHD as a Serious Risk Factor for Early Smoking and Nicotine Dependence in Adulthood; Swantje Matthies, Sebastian Holzner, Bernd Feige, Corinna Scheel, Evgeniy Perlov, Dieter Ebert, Ludger Tebartz van Elst, and Alexandra Philipsen

136. Examination of the Diurnal Assumptions of the Test of Variables of Attention for Elementary Students; David P. Hurford, Kara A. Lasater, Sara E. Erickson and Nicole E. Kiesling

137. Validation of the Chinese Strengths and Weaknesses of ADHD-Symptoms and Normal-Behaviors Questionnaire in Hong Kong; Kelly Y. C. Lai, Patrick W. L. Leung, Ernest S. L. Luk, Ann S. Y. Wong, Lawrence S. C. Law, and Karen K. Y. Ho

138. The ASRS-6 Has Two Latent Factors: Attention Deficit and Hyperactivity; Morten Hesse

139. Environmental Stimulation Does Not Reduce Impulsive Choice in ADHD: A Pink Noise Study; Baris Metin, Herbert Roeyers, Jan R. Wiersema, Jaap J. van der Meere, Roos Gasthuys and Edmund Sonuga-Barke

140. An Open-Label, Randomized Trial of Methylphenidate and Atomoxetine Treatment in Adults With ADHD; Hsing-Chang Ni, Yu-Ju Lin, Susan Shur-Fen Gau, Hui-Chun Huang and Li-Kuang Yang

141. An Empirical Evaluation of ADHD Coaching in College Students; Frances Prevatt and Sherry Yelland

142. Behavioral and Academic Progress of Children Displaying Substantive ADHD Behaviors in Special Education: A 1-Year Follow-up; Regina Stoutjesdijk, Evert M. Scholte and Hanna Swaab

143. No Evidence for Inhibitory Deficits or Altered Reward Processing in ADHD: Data From a New Integrated Monetary Incentive Delay Go/No-Go Task; Ellen Demurie, Herbert Roeyers, Jan R. Wiersema and Edmund Sonuga-Barke

144. Treating Comorbid Anxiety in Adolescents With ADHD Using a Cognitive Behavior Therapy Program Approach; Stephen Houghton, Nadiyah Alsalmi, Carol Tan, Myra Taylor, and Kevin Durkin

145. Oppositional Defiant Disorder in Adults With ADHD; Frederick W. Reimherr, Barrie K. Marchant,John L. Olsen,Paul H. Wender and Reid J. Robison

146. Self-Concept of College Students With ADHD: Discordance Between Self- and Parent-Reports; Jason M. Nelson

147. Differential Response to Methylphenidate in Inattentive and Combined Subtype ADHD; Susan H. Beery, Herbert C. Quay and William E. Pelham, Jr.

American Journal of Drug and Alcohol Abuse

1. Resting state synchrony in anxiety-related circuits of abstinent alcohol-dependent patients Csaba Orban, John McGonigle, Nicola J. Kalk, David Erritzoe, Adam D. Waldman, David J. Nutt, Eugenii A. Rabiner, Anne R. Lingford-Hughes

2. Functional connectivity and cannabis use in high-risk adolescents Jon M. Houck, Angela D. Bryan, Sarah W. Feldstein Ewing
3. Associations between fractional anisotropy and problematic alcohol use in juvenile justice-involved adolescents Rachel E. Thayer, Tiffany J. Callahan, Barbara J. Weiland, Kent E. Hutchison, Angela D. Bryan
4. Occupation as an Independent Risk Factor for Binge Drinking Andrew James Barnes, , E. Richard Brown
5. Family history of alcohol use disorders and neuromaturation: a functional connectivity study with adolescents Andrea D. Spadoni, Alan N. Simmons, Tony T. Yang, Susan F. Tapert
6. Effects of alcohol use initiation on brain structure in typically developing adolescents Monica Luciana, Paul F. Collins, Ryan L. Muetzel, Kelvin O. Lim
7. Gender disparity in utilization rates of substance abuse services among female ex-offenders: a population-based analysis Senik T. Mahmood, Michael G. Vaughn, Michael Mancini, Q. John Fu
8. Further evaluation of the construct, convergent and criterion validity of the Gambling Urge Scale with university-student gamblers Lisham Ashrafioun, Harold Rosenberg, Nicole A. Cross, Thomas J. Brian
9. Validating the ability of a single-item assessing drunkenness to detect hazardous drinking Adam E. Barry, Beth H. Chaney, Michael L. Stellefson, Virginia Dodd
10. The impact of twelve-step program familiarity and its in-session discussion on counselor credibility Cory B. Dennis, Brian D. Roland, Barry Loneck
11. A proposal to evaluate mechanistic efficacy of hallucinogens in addiction treatment Brittany Vasae Burdick, Bryon Adinoff
12. The advent of a new pseudoephedrine product to combat methamphetamine abuse Albert W. Brzeczko, Ronald Leech, Jeffrey G. Stark
13. Marijuana use is associated with risky sexual behaviors in treatment-seeking polysubstance abusers Leonardo F. Andrade, Kathleen M. Carroll, Nancy M. Petry
14. The mediating effect of self-esteem, depression and anxiety between satisfaction with body appearance and problematic internet use Beatrix Koronczai, Gyngyi Kknyei, Rbert Urbn, Bernadette Kun, Orsolya Ppay, Katalin Nagygyrgy, Mark D. Griffiths, Zsolt Demetrovics
15. Effectiveness and feasibility study of routine HIV rapid testing in an urban methadone maintenance treatment program Randy Seewald, R. Douglas Bruce, Rashiah Elam, Ruy Tio, Sara Lorenz, Patricia Friedmann, David Rabin, Yana B. Garger, Valentin Bonilla Jr., David C. Perlman
16. Factors associated with high-frequency illicit methadone use among rural Appalachian drug users Martin T. Hall, Carl G. Leukefeld, Jennifer R. Havens
17. Preliminary evaluation of a model of stimulant use, oxidative damage and executive dysfunction Theresa Winhusen, Jessica Walker, Gregory Brigham, Daniel Lewis, Eugene Somoza, Jeff Theobald, Veronika Somoza

18. APOE e4 genotype and cigarette smoking in adults with normal cognition and mild cognitive impairment: a retrospective baseline analysis of a national dataset Raj K. Kalapatapu, Kevin L. Delucchi

19. High and Low Neurobehavior Disinhibition Clusters within Locales: Implications for Community Efforts to Prevent Substance Use Disorder Ty A. Ridenour, Maureen Reynolds, Ola Ahlqvist, Zu Wei Zhai, Levent Kirisci, Michael M. Vanyukov, Ralph E. Tarter

20. Changes in Smoking for Adults with and without Alcohol and Drug Use Disorders: Longitudinal Evaluation in the US Population Andrea H. Weinberger, Corey E. Pilver, Rani A. Hoff, Carolyn M. Mazure, Sherry A. McKee

21. Longitudinal Modeling of Transmissible Risk in Boys Who Subsequently Develop Cannabis Use Disorder Levent Kirisci, Ralph E. Tarter, Ty Ridenour, Maureen Reynolds, Michael Vanyukov

22. Probability and Predictors of Transition from Abuse to Dependence on Alcohol, Cannabis, and Cocaine: Results from the National Epidemiologic Survey on Alcohol and Related Conditions Ludwing Flrez-Salamanca, Roberto Secades-Villa, Deborah S. Hasin, Linda Cottler, Shuai Wang, Bridget F. Grant, Carlos Blanco

23. The Multiple Bottle Effect Is Overridden in Male and Female Rats by Simultaneous Presentation of Two Oral Nicotine Solutions Swapnali Halder, Jeffrey M. Lynch, Amy R. Pearce

24. Naltrexone (50 mg) Plus Psychotherapy in Alcohol-Dependent Patients: A Meta-Analysis of Randomized Controlled Trials Joanna Jarosz, Katarzyna Miernik, Maria Wchal, Jacek Walczak, Gnther Krumpl

25. Physician Detection of Unhealthy Substance Use on Inpatient Teaching and Hospitalist Medical Services Stephen R. Holt, , Jorge Ramos, , Michael Harma, , Felix Cabrera, , Coeurlida Louis-Ashby, , An Dinh, , David A. Fiellin, , Jeanette M. Tetrault,

26. Physical Activity and Alcohol Use Disorders Nadra E. Lisha, , Steve Sussman, , FAAHB, FAPA, Adam M. Leventhal,

27. Trends in Opioid Agonist Therapy in the Veterans Health Administration: Is Supply Keeping up with Demand? Elizabeth M. Oliva, Jodie A. Trafton, Alex H.S. Harris, Adam J. Gordon

28. Perceptions of HIV Risk among Methamphetamine Users in China Brian C. Kelly, , Tieqiao Liu, , Guanbai Zhang, Wei Hao, Jichuan Wang

29. Resisting the Urge to Smoke and Craving during a Smoking Quit Attempt on Varenicline: Results from a Pilot FMRI Study Karen J. Hartwell, Todd LeMatty, Aimee L. McRae-Clark, Kevin M. Gray, Mark S. George, Kathleen T. Brady

30. Opioid-Like Effects of the Neurokinin 1 Antagonist Aprepitant in Patients Maintained on and Briefly Withdrawn from Methadone Jermaine D. Jones, Taylor Speer, Sandra D. Comer, Stephen Ross, John Rotrosen, Malcolm S. Reid

31. Pharmacokinetic Interactions between Buprenorphine/Naloxone and Raltegravir in Subjects Receiving Chronic Buprenorphine/Naloxone Treatment R. Douglas Bruce, David E. Moody, Diane Chodkowski, Laurie Andrews, Wenfang B. Fang, Jerdravee Morrison, Theresa L. Parsons, Gerald H. Friedland

32. Aripiprazole in an Animal Model of Chronic Alcohol Consumption and Dopamine D2 Receptor Occupancy in Rats Ramakrishna Nirogi, , Vishwottam Kandikere, , Pradeep Jayarajan, , Gopinadh Bhyrapuneni, , Ramanatha Saralaya, Nageswararao Muddana, Renny Abraham

33. Client and Program Characteristics Associated with Wait Time to Substance Abuse Treatment Entry Christina M. Andrews, Hee-Choon Shin, Jeanne C. Marsh, Dingcai Cao

34. Does Alcohol Craving Mediate the ImpulsivityAggression Relationship in Recently Detoxified Alcohol-Dependent Patients? H. G. Roozen, B. J. M. van de Wetering, I. H. A. Franken

35. The Social Support and Social Network Characteristics of Smokers in Methadone Maintenance Treatment Marcel Alejandro de Dios, Cassandra A. Stanton, Celeste M. Caviness, Raymond Niaura, Michael Stein

36. Social Contexts of Drinking and Subsequent Alcohol Use Disorder Among College Students Kenneth H. Beck, , Kimberly M. Caldeira, , Kathryn B. Vincent, , Amelia M. Arria,

37. Interpersonal Guilt in College Student Pathological Gamblers Geoffrey W. Locke, Robert Shilkret, Joyce E. Everett, Nancy M. Petry

38. Substance Abuse Treatment Readmission Patterns of Asian Americans: Comparisons with Other Ethnic Groups Jiang Yu, , Lynn A. Warner,

39. EPHX1 Gene Polymorphisms in Alcohol Dependence and their Distribution among the Indian Populations L. V. K. S. Bhaskar, K. Thangaraj, Minarbha Patel, Anish M. Shah, K. Gopal, L. Saikrishna, Rakesh Tamang, Lalji Singh, V. R. Rao

40. Correlates of Concurrent Energy Drink and Alcohol Use among Socially Active Adults Brooke E. Wells, Brian C. Kelly, , Mark Pawson, Amy LeClair, Jeffrey T. Parsons, Sarit A. Golub

2 Rejection of linear regression on basis of correct assumptions

International Psychogeriatrics

1. Which older adults maintain benefit from cognitive training? Use of signal detection methods to identify long-term treatment gains; J. K. Fairchild, L. Friedman, A. C. Rosen and J. A. Yesavage

3 Rejection of linear regression on basis of not meeting incorrect assumptions

4 Correct linear regression

International Psychogeriatrics

1. Psychosocial correlates of nutritional status of family caregivers of persons with dementia; Laetitia Rullier, Alexia Lagarde, Jean Bouisson, Valrie Bergua, Marion Torres and Pascale Barberger-Gateau
2. Affective functioning after delirium in elderly hip fracture patients; Chantal J. Slor, Joost Witlox, Ren W. M. M. Jansen, Dimitrios Adamis, David J. Meagher, Esther Tieken, Alexander P. J. Houdijk, Willem A. van Gool, Piet Eikelenboom and Jos F. M. de Jonghe
3. The importance of the supervisor for the mental health and work attitudes of Australian aged care nurses; John Rodwell and Angela Martin

5 Mentioned all correct assumptions but not if the normality assumption was tested on the residuals or on X or Y

International Psychogeriatrics

1. The neuropsychological sequelae of delirium in elderly patients with hip fracture three months after hospital discharge; Joost Witlox, Chantal J. Slor, Ren W.M.M. Jansen, Kees J. Kalisvaart, Mireille F.M. van Stijn,Alexander P.J. Houdijk, Piet Eikelenboom, Willem A. van Gool, and Jos F.M. de Jonghe
2. Factors affecting subjective memory complaints in the AIBL aging study: biomarkers, memory, affect, and age; R. Buckley, M. M. Saling, D. Ames, C. C. Rowe, N. T. Lautenschlager, S. L. Macaulay, R. N. Martins, C. L. Masters, T. OMeara, G. Savage, C. Szoeke, V. L. Villemagne, K. A. Ellis and Australian Imaging Biomarkers and Lifestyle Study of Aging (AIBL) Research Group
3. Factors associated with depressive symptoms in older Taiwanese adults in a long-term care community; Ya-Chuan Hsu, Terry Badger, Pamela Reed and Elaine Jones
4. Decision-making deficits in normal elderly persons associated with executive personality disturbances; Christopher M. Nguyen, Joseph Barrash, Anna L. Koenigs, Antoine Bechara, Daniel Tranel and Natalie L. Denburg

Journal of Attention Disorders

1. An Examination of Prescription Stimulant Misuse and Psychological Variables Among Sorority and Fraternity College Populations; Crystal L. Dussault and Lisa L. Weyandt

6 Did not test all but some correct assumptions, included neither normality of variables nor residuals

International Psychogeriatrics

1. Longitudinal changes in functional disability in Alzheimer's disease patients; H. Michael Arrighi, Isabelle Glinas, Trent P. McLaughlin, Jacqui Buchanan and Serge Gauthier
2. Adaptation and analysis of psychometric features of the Caregiver Risk Screen: a tool for detecting the risk of burden in family caregivers; Silvia Martinez-Rodriguez, Nuria Ortiz-Marqus, Ioseba Iraurgi, Mara Carrasco and Jos J. Miguel

3. Quality of life (QOL) of older adult community choral singers in Finland; Julene K. Johnson, Jukka Louhivuori, Anita L. Stewart, Asko Tolvanen, Leslie Ross and Pertti Era

4. ADHD symptoms across the lifespan in a population-based Swedish sample aged 65 to 80; Taina Guldberg-Kjr, Sally Sehlin and Boo Johansson

5. The course of neuropsychiatric symptoms in nursing-home patients with dementia over a 53-month follow-up period; Geir Selbk, Knut Engedal, Jrat altyt Benth and Sverre Bergh

Journal of Attention Disorders

1. Acute Effects of MPH on the ParentTeen Interactions of Adolescents With ADHD William E. Pelham, Jr., David L. Meichenbaum, Bradley H. Smith, Margaret H. Sibley, Elizabeth M. Gnagy and Oscar Bukstein

2. A Decade of EEG Theta/Beta Ratio Research in ADHD: A Meta-Analysis; Martijn Arns, C. Keith Conners and Helena C. Kraemer

American Journal of Drug and Alcohol Abuse

1. Altered resting-state connectivity in adolescent cannabis users Catherine Orr, Rowen Morioka, Brendan Behan, Sameer Datwani, Marika Doucet, Jelena Ivanovic, Clare Kelly, Karen Weierstall, Richard Watts, Bobby Smyth, Hugh Garavan

7 Use of linear regression but no indication if any or which assumptions were tested

International Psychogeriatrics

1. Sense of coherence and pain experience in older age; Ulrich Wiesmann, Jessie Dezutter and Hans-Joachim Hannich

2. Stability and change in level of probable depression and depressive symptoms in a sample of middle and older-aged adults ; Richard A. Burns, Peter Butterworth, Mary Luszcz and Kaarin J. Anstey

3. Direct costs associated with depressive symptoms in late life: a 4.5-year prospective study; Melanie Luppa, Hans-Helmut Knig, Dirk Heider, Hanna Leicht, Tom Motzek, Georg Schomerus and Steffi G. Riedel-Heller

4. Sense of coherence, burden, and affective symptoms in family carers of people with dementia; Vasiliki Orgeta and Elena Lo Sterzo

5. Relationship between delirium and behavioral symptoms of dementia; Philippe Landreville, Philippe Voyer and Pierre-Hugues Carmichael

6. Progression of Alzheimers disease during a three-year follow-up using the CERAD-NB total score: Kuopio ALSOVA study; Ilona Hallikainen, Tuomo Hnninen, Mikael Fraunberg, Kristiina Hongisto,Tarja Vlimki, Asta Hiltunen, Pertti Karppi, Juhani Sivenius, Hilkka Soininen, Anne M. Koivisto for the ALSOVA study group

7. Neural correlates of the components of the clock drawing test; Teruyuki Matsuoka, Jin Narumoto, Aiko Okamura, Shogo Taniguchi, Yuka Kato, Keisuke Shibata, Kaeko Nakamura, Chio Okuyama, Kei Yamada and Kenji Fukui

8. Caregiver rating bias in mild cognitive impairment and mild Alzheimer's disease: impact of caregiver burden and depression on dyadic rating discrepancy across domains; Livia Pfeifer, Reinhard Drobetz, Sonja Fankhauser, Moyra E. Mortby, Andreas Maercker and Simon Forstmeier

9. Burden of caregivers for patients with mild cognitive impairment in Japan; Satoshi Hayashi, Seishi Terada, Shigeto Nagao, Chikako Ikeda, Aki Shindo, Etsuko Oshima, Osamu Yokota and Yosuke Uchitomi

10. Smell identification function as a severity and progression marker in Alzheimer's disease; Latha Velayudhan, Megan Pritchard, John F. Powell, Petroula Proitsi and Simon Lovestone

11. Quality of life in dementia: the role of non-cognitive factors in the ratings of people with dementia and family caregivers; Maria Fernanda Barroso Sousa, Raquel Luiza Santos, Cynthia Arcoverde, Pedro Simes, Tatiana Belfort, Isabel Adler, Camila Leal and Marcia Cristina Nascimento Dourado

12. Factors associated with quality of life in dementia patients in long-term care; Mara Crespo, Carlos Hornillos and Mnica Bernaldo de Quirs

13. Does tip-of-the-tongue for proper names discriminate amnestic mild cognitive impairment? Onsimo Juncos-Rabadn, David Facal, Cristina Lojo-Seoane and Arturo X. Pereiro

14. Engagement in social activities and progression from mild to severe cognitive impairment: the MYHAT study; Tiffany F. Hughes, Jason D. Flatt, Bo Fu, Chung-Chou H. Chang and Mary Ganguli

15. Residents with mentalphysical multimorbidity living in long-term care facilities: prevalence and characteristics. A systematic review; Anne M. A. van den Brink, Debby L. Gerritsen, Richard C. Oude Voshaar and Raymond T. C. M. Koopmans

16. Sensitivity to change of composite and frequency scores of the Neuropsychiatric Inventory in mild to moderate dementia; Jeffrey L. Cummings, Ralf Ihl, Horst Herrschaft, Robert Hoerr and Michael Tribanek

17. Sense of community in long-term care: the views of family caregivers of elderly military veterans; Ana Petrovic-Poljak and Candace Konnert

18. The Hospital Anxiety and Depression Scale: low sensitivity for depression screening in demented and non-demented hospitalized elderly; Nikolaos Samaras, Franois R. Herrmann, Dimitrios Samaras, Pierre-Olivier Lang, Alessandra Canuto, Alexandre Forster, Henriette Hilleret and Gabriel Gold

19. Characterizing spontaneously reported cognitive complaints: the development and reliability of a classification instrument; Daniel Apolinario, Rafaela Branco Miranda, Claudia Kimie Suemoto, Regina Miksian Magaldi, Alexandre Leopold Busse, Aline Thomaz Soares, Leonardo da Costa Lopes, Juliana Yumi Tizon Kasai, Erika Satomi, Elina Lika Kikuchi and Wilson Jacob-Filho

20. Flexible and tenacious goal pursuit lead to improving well-being in an aging population: a ten-year cohort study; Rebecca E. Kelly, Alex M. Wood and Warren Mansell

21. Altered neurochemical metabolites in Alzheimer's disease patients with unawareness of deficits; Yi-Chun Yeh, Cheng-Fang Yen, Chun-Wei Li, Yu-Ting Kuo, Chia-Hui Chen, Chen-Chang Lee, Gin-Chung Liu, Mei-Feng Huang, Tai-Ling Liu and Cheng-Sheng Chen

22. Path analysis of suicide ideation in older people; Seolmin Kim, Jee Hyun Ha, Jaehak Yu, Doo-Heum Park and Seung-Ho Ryu

23. The impact of forced displacement in World War II on mental health disorders and health-related quality of life in late life a German population-based study; Simone Freitag, Elmar Braehler, Silke Schmidt and Heide Glaesmer

24. Factors associated with quality of life among family members of patients with dementia in Cyprus; Evridiki Papastavrou, Panayiota Andreou, Nicos Middleton, Savvas Papacostas and Irini Kyriacou Georgiou

25. The impact of neuropsychiatric symptoms on caregiver distress and quality of life in persons with dementia in an Asian tertiary hospital memory clinic; S. A. Khoo, T. Y. Chen, Y. H. Ang and P. Yap

26. Montreal Cognitive Assessment and Mini-Mental State Examination performance in patients with mild-to-moderate dementia with Lewy bodies, Alzheimer's disease, and normal participants in Taiwan; Carol Sheei-Meei Wang, Ming-Chyi Pai, Pai-Lien Chen, Nien-Tsen Hou, Pei-Fang Chien and Ying-Che Huang

27. Speech and orofacial apraxias in Alzheimers disease; Maysa Luchesi Cera,Karin Zazo Ortiz, Paulo Henrique Ferreira Bertolucci, and Thas Soares Cianciarullo Minett

28. Distinct cognitive phenotypes in Alzheimers disease in older people; Emma R.L.C. Vardy, Andrew H. Ford, Peter Gallagher, Rosie Watson, Ian G. McKeith, Andrew Blamire and John T. OBrien

29. Health, body image, gender, and migration status: their relationship to sexuality in old age; Darya Shkolnik and Esther Iecovich

30. Covariant perfusion patterns provide clues to the origin of cognitive uctuations and attentional dysfunction in Dementia with Lewy bodies; John-Paul Taylor, Sean J. Colloby, Ian G. McKeith and John T. OBrien

Journal of Attention Disorders

1. Conceptual Structure of the Symptoms of Adult ADHD According to the DSM-IV and Retrospective Wender-Utah Criteria; Angelika Glckner-Rist, Anya Pedersen and Fred Rist

2. Marital and Coparenting Relationships: Associations With Parent and Child Symptoms of ADHD; David Williamson and Charlotte Johnston

3. The SWAN Captures Variance at the Negative and Positive Ends of the ADHD Symptom Dimension; Anne B. Arnett, Bruce F. Pennington, Angela Friend, Erik G. Willcutt, Brian Byrne, Stefan Samuelsson, and Richard K. Olson

4. Sluggish Cognitive Tempo Among Young Adolescents With ADHD: Relations to Mental Health, Academic, and Social Functioning; Stephen P. Becker and Joshua M. Langberg

5. The Direct Effects of Inattention and Hyperactivity/Impulsivity on Peer Problems and Mediating Roles of Prosocial and Conduct Problem Behaviors in a Community Sample of Children; Brendan F. Andrade and Rosemary Tannock

6. The Relationship Between Executive Functions and Quality of Life in Adults With ADHD; Adi Stern, Yehuda Pollak, Omer Bonne, Elad Malik, and Adina Maeir

7. Cognitive Processes in ADHD and Aspergers Disorder: Overlaps and Differences in PASS Profiles; Stefano Taddei and Bastianina Contena

8. Sex- and Subtype-Related Differences in the Comorbidity of Adult ADHDs Silke Gro-Lesch, Astrid Dempfle, Susanne Reichert, Thomas Jans, Julia Geissler, Sarah Kittel-Schneider, Thuy Trang Nguyen, Andreas Reif, Klaus-Peter Lesch and Christian Peter Jacob

9. Increased Erythrocyte Eicosapentaenoic Acid and Docosahexaenoic Acid Are Associated With Improved Attention and Behavior in Children With ADHD in a Randomized Controlled Three-Way Crossover Trial; Catherine M. Milte, Natalie Parletta, Jonathan D. Buckley, Alison M. Coates, Ross M. Young and Peter R. C. Howe

10. Personality Traits and Comorbidity in Adults With ADHD Johanne Telnes Instanes, Jan Haavik and Anne Halmy

11. A Double Dissociation Between Inattentive and Impulsive Traits, on Tasks of Visual Processing and Emotion Regulation; Dorit Ben Shalom, Ziv Ronel, Yifat Faran, Gal Meiri, Lidia Gabis and Kimberly A. Kerns

12. Evaluating Parental Disagreement in ADHD Diagnosis: Can We Rely on a Single Report From Home? Arthur Caye, Julia D. Machado and Lus A. Rohde

13. Adults With ADHD-Without Insomnia History Have Subclinical Sleep Disturbance but Not Circadian Delay: An ADHD Phenotype? Rachel E. Fargason, Abigail Fernandez Hollar, Samantha White and Karen L. Gamble

14. Neurocognitive and Behavioral Predictors of Math Performance in Children With and Without ADHD; Tanya N. Antonini, Kathleen M. Kingery, Megan E. Narad, Joshua M. Langberg, Leanne Tamm and Jeffery N. Epstein

15. The Relation Between ADHD Symptoms and Alcohol Use in College Students; Glenn R. Mesman

16. An Analysis of the Barkley Deficits in Executive Functioning Scale in a College Population: Does It Predict Symptoms of ADHD Better Than a Visual-Search Task? Vincent Malik Dehili, Frances Prevatt and Theodora P. Coffman

17. Differential Effect of Cognitive Training on Executive Functions and Reading Abilities in Children With ADHD and in Children With ADHD Comorbid With Reading Difficulties; Tzipi Horowitz-Kraus

18. Gambling, Delay, and Probability Discounting in Adults With and Without ADHD; Zhijie Dai, Sarah-Eve Harrow, Xianwen Song, Julia J. Rucklidge and Randolph C. Grace

19. Movement Scaling in Children With ADHD-Combined Type; Rebecca A. Langmaid, Nicole Papadopoulos, Beth P. Johnson, James Phillips and Nicole J. Rinehar

20. The Role of Emotional Distress and ADHD on Institutional Behavioral Disturbance and Recidivism Among Offenders; Rafael A. Gonzlez, Gisli H. Gudjonsson, June Wells and Susan Young

21. Neonatal Sleep Predicts Attention Orienting and Distractibility; Ronny Geva, Hagit Yaron and Jacob Kuint

22. Executive Functioning in Children and Adolescents With Symptoms of Sluggish Cognitive Tempo and ADHD; Eva Angelina Araujo Jimnez, Mara Claustre Jan Ballabriga, Albert Bonillo Martin, Francisco Javier Arrufat, and Rodrigo Serra Giacobo

23. Congruent Validity of the Strengths and Difficulties Questionnaire to Screen for Comorbidities in Children With ADHD; Joshua Bekker, Dorothy Bruck and Emma Sciberras

24. The Relation of Poor Emotional Awareness and Externalizing Behavior Among Children With ADHD; Perry I. Factor Paul J. Rosen and Rachel A. Reyes

25. One Size Fits All? Slow Cortical Potentials Neurofeedback: A Review; Kerstin Mayer, Sarah N. Wyckoff and Ute Strehl

26. Health Care Costs Associated With Parent- Reported ADHD: A Longitudinal Australian PopulationBased Study; Emma Sciberras, Nina Lucas, Daryl Efron, Lisa Gold, Harriet Hiscock and Jan M. Nicholson

27. Comparison of Three ADHD Screening Instruments in College Students of Varying Cognitive Ability; Melissa D. Fuller-Killgore, Jonathan Burlison and William Dwyer

28. The Positive Illusory Bias in Children and Adolescents With ADHD: Further Evidence; Eftychia Volz-Sidiropoulou, Maren Boecker and Siegfried Gauggel

29. A Longitudinal Examination of the Developmental Executive Function Hierarchy in Children With Externalizing Behavior Problems; Carin Tillman, Karin C. Brocki, Lin Srensen and Astri J. Lundervold

30. Distinct ADHD Symptom Clusters Differentially Associated With Personality Traits; Ashley A. McKinney, Will H. Canu and H. G. Schneider

31. Comorbidity and Phenomenology of Bipolar Disorder in Children With ADHD; Eduardo Serrano, Lourdes Ezpeleta and Josefina Castro-Fornieles

32. Associations Between Sleep Characteristics, Seasonal Depressive Symptoms, Lifestyle, and ADHD Symptoms in Adults; Denise Bijlenga, Kristiaan B. van der Heijden, Minda Breuk, Eus J. W. van Someren, Maria E. H. Lie, A. Marije Boonstra, Hanna J. T. Swaab, and J. J. Sandra Kooij

33. Quality of Life in Adults Aged 50+ With ADHD; Michael B. Lensing, Pl Zeiner, Leiv Sandvik and Stein Opjordsmoen

34. Neuropsychological Deficits Are Not Predictive of Deficient Emotional Self- Regulation in Adults With ADHD; Craig B. H. Surman, Joseph Biederman, Thomas Spencer, Carolyn A. Miller, Carter R. Petty and Stephen V. Faraone

35. Determining the Accuracy of Self-Report Versus Informant-Report Using the Conners Adult ADHD Rating Scale Lisa Alexander and Laura Liljequist

36. Positive and Negative Affect in Clinic- Referred Youth With ADHD; Izumi Okado, Charles W. Mueller and Brad J. Nakamura

37. The Impact of Early Classroom Inattention on Phonological Processing and Word-Reading Development; Cassandra K. Dittman

38. ADHD Symptoms and Academic Adjustment to College: The Role of Parenting Style; Heather A. Jones, Annie E. Rabinovitch and Rebecca R. Hubbard

39. Functional Impairment Mediates the Relationship Between Adult ADHD Inattentiveness and Occupational Outcome; Bjrn Gjervan, Odin Hjemdal and Hans M. Nordahl

40. Differentiating Behavioral Ratings of Inattention, Impulsivity, and Hyperactivity in Children: Effects on Reading Achievement; Andy V. Pham

41. Cognitive Responses to Stress, Depression, and Anxiety and Their Relationship to ADHD Symptoms in First Year Psychology Students; Sandra J. Alexander and Allyson G. Harrison

42. Pre- and Postnatal Risk Factors for ADHD in a Nonclinical Pediatric Population; Sharon K. Sagiv, Jeff N. Epstein, David C. Bellinger and Susan A. Korrick

43. Risk Screening for ADHD in a College Population: Is There a Relationship With Academic Performance? Jonathan D. Burlisonand William O. Dwyer

44. Is ADHD a Risk Factor Independent of Conduct Disorder for Illicit Substance Use?: A Meta-Analysis and Meta-regression Investigation Maria Antonia Serra-Pinheiro, Evandro S. F. Coutinho, Isabella S. Souza, Camilla Pinna, Ddia Fortes, Catia Arajo, Claudia M. Szobot, Luis A. Rohde, and Paulo Mattos

45. Cognitive Deficits in Adults With ADHD Go Beyond Comorbidity Effects Katiane L. Silva1, Paula O. Guimares-da-Silva, Eugenio H. Grevet1, Marcelo M. Victor, Carlos A. I. Salgado, Eduardo S. Vitola, Nina R. Mota, Aline G. Fischer, Vernica Contini, Felipe A. Picon1, Rafael G. Karam1, Paulo Belmonte-de-Abreu1, Luis A. Rohde, and Claiton H. D. Bau

46. Working Memory and Response Inhibition as One Integral Phenotype of Adult ADHD? A Behavioral and Imaging Correlational Investigation; Marcel Romanos, Christian Jacob, Paul Pauli and Andreas J. Fallgatter Martin Schecklmann, Ann-Christine Ehlis, Michael M. Plichta, Thomas Dresler, Monika Heine, Andrea Boreatti-Hmmer

American Journal of Drug and Alcohol Abuse

1. Sex differences in psychosocial correlates of concurrent substance use among heterosexual, homosexual and bisexual college students Gillian L. Schauer, Carla J. Berg, Lawrence O. Bryant

2. Striatal-insula circuits in cocaine addiction: implications for impulsivity and relapse risk Meredith J. McHugh, Catherine H. Demers, Jacquelyn Braud, Richard Briggs, Bryon Adinoff, Elliot A. Stein

3. An intrinsic connectivity network approach to insula-derived dysfunctions among cocaine users Krista M. Wisner, Edward H. Patzelt, Kelvin O. Lim, Angus W. MacDonald III

4. A preliminary investigation of Stroop-related intrinsic connectivity in cocaine dependence: associations with treatment outcomes Marci R. Mitchell, Iris M. Balodis, Elise E. DeVito, Cheryl M. Lacadie, Jon Yeston, Dustin Scheinost, R. Todd Constable, Kathleen M. Carroll, Marc N. Potenza

5. Functional connectivity in inhibitory control networks and severity of cannabis use disorder Francesca Filbey, Uma Yezhuvath

6. Do drinking games matter? An examination by game type and gender in a mandated student sample Jacqueline Alfonso, Spencer D. Deschenes

7. Therapist predictors of treatment delivery fidelity in a community-based trial of 12-step facilitation Barbara K. Campbell, Allison Buti, Holly E. Fussell, Priya Srikanth, Dennis McCarty, Joseph R. Guydish

8. QTc interval prolongation for patients in methadone maintenance treatment: a five years follow-up study Ayman Fareed, Sreedevi Vayalapalli, Kelly Scheinberg, Robin Gale, Jennifer Casarella, Karen Drexler

9. Sleep Quality Moderates the Relation between Depression Symptoms and Problematic Cannabis Use among Medical Cannabis Users Kimberly A. Babson, Matthew Tyler Boden, Marcel O. Bonn-Miller

10. Impulsivity, Expectancies, and Evaluations of Expected Outcomes as Predictors of Alcohol Use and Related Problems Eugene M. Dunne, Jonathan Freedlander, Kimberly Coleman, Elizabeth C. Katz

11. The Impact of Alcohol on Health-Related Quality of Life in Belgrade University Students Darija Kisic-Tepavcevic, Tatjana Gazibara, Aleksandra Popovic, Goran Trajkovic, Tatjana Pekmezovic

12. Functional Social Support within a Medical Supervised Outpatient Treatment Program Jochen Mutschler, Sarah Eifler, Glseren Dirican, Martin Grosshans, Falk Kiefer, Wulf Rssler, Alexander Diehl

13. The Relationship between Wisdom and Abstinence Behaviors in Women in Recovery from Substance Abuse Julia A. DiGangi, Leonard A. Jason, Leslie Mendoza, Steve A. Miller, Richard Contreras

8 Assumed/tested normally distributed X but not the normality of the residuals

Journal of Attention Disorders

1. The Relationship Between ADHD Symptoms, Mood Instability, and Self-Reported Offending; Gisli H. Gudjonsson, Jon Fridrik Sigurdsson, Tomas F. Adalsteinsson and Susan Young

2. Differentiating Co-Occurring Behavior Problems in Children With ADHD: Patterns of Emotional Reactivity and Executive Functioning; Paulo A. Graziano, Joseph P. McNamara, Gary R. Geffken and Adam M. Reid

9 Assumed/tested normally distributed Y but not the normality of the residuals

10 Assumed/tested normally distributed X and Y but not the normality of the residuals

11 Assumed/tested normally distributed variables but did not indicate if X or Y or both and did not test the normality of the residuals

12 Other misconceptions about assumptions

International Psychogeriatrics

1. Quality of dying of nursing home residents with dementia as judged by relatives; Nicole van Uden, Lieve Van den Block, Jenny T. van der Steen, Bregje D. Onwuteaka-Philipsen, An Vandervoort, Robert Vander Stichele and Luc Deliens on behalf of EURO IMPACT
2. Life satisfaction and frailty in community-based older adults: cross-sectional and prospective analyses; Philip D. St John, Suzanne L. Tyas and Patrick R. Montgomery

Journal of Attention Disorders

1. Do Symptoms of Sluggish Cognitive Tempo in Children With ADHD Symptoms Represent Comorbid Internalizing Difficulties? Annie A. Garner, Sylvie Mrug, Bart Hodgens and Cryshelle Patterson

Part III

Q3 top 3: Zeitschrift fur Klinische Psychologie und Psychotherapie, Journal of Obsessive-Compulsive and Related Disorders , International Journal of Psychology and Psychological Therapy

1 No Model of interest

Zeitschrift fur Klinische Psychologie und Psychotherapie

1. Krankheitskosten der Borderline Persnlichkeitsstrung aus gesellschaftlicher Perspektive; Till Wagner, Stefan Roepke, Paul Marschall, Christian Stiglmayr, Babette Renneberg, Dieter Gieb, Claudia Dambacher, Sara Matthies, Harriet Salbach-Andrae, Steffen Flea und Thomas Fydrich

2. Psychische Strungen und individuelle Lebensqualitt bei der chronisch progredient-terminalen Erkrankung Amyotrophe Lateralsklerose (ALS) Nonnenmacher, S., Hammer, E. M.2, Lul, D., Hautzinger, M. und Kbler, A.
3. Remission, Response und deren Prdiktion nach einer DialektischBehavioralen Therapie der Borderline-Persnlichkeitsstrung im stationren Setting Christoph Krger, Susanne Harbeck, Imke Rickert, Eileen Wollburg, Katrin Gersch, Michael Armbrust und Sren Kliem
4. Entwicklung und Evaluation des Fragebogens zu belastenden Sozialerfahrungen in der Peergroup (FBS) Lisa Sansen, Benjamin Iffland, Claudia Catani und Frank Neuner
5. Das Vancouver Obsessional Compulsive Inventory Revised (VOCI-R) Ist sein Einsatz in der Diagnostik und Therapieplanung bei Zwangspatienten sinnvoll? Sascha Gnner, Johanna Schmid, Stefanie Gnner, Rainer Leonhart und Willi Ecker
6. Ressourcentagebuch: Verbesserung der Emotionsregulation und der Ressourcenrealisierung durch therapeutisches Schreiben im Anschluss an eine Psychotherapie Eine Pilotstudie; Anne Katrin Risch und Gabriele Wilz
7. Erziehungstraining fr Eltern mit einer psychischen Erkrankung eine Pilotstudie; Olga Propp, Miriam Mller und Sren Kliem
8. Ziel erreicht! Aber auch verndert? Zwei basale Perspektiven in der Psychotherapieerfolgsbeurteilung; Dominik lsmann und Dietmar Schulte
9. Kognitive Therapie bei krperdysmorpher Strung; Viktoria Ritter und Ulrich Stangier
10. Body Dysmorphic Disorder: Neurobiological Features and an Updated Model; Wei Li, Donatello Arienzo, and Jamie D. Feusner
11. Krperbildstrungen bei krperdysmorpher Strung und Essstrungen Wo bestehen Unterschiede und wo bestehen Gemeinsamkeiten? Ines Kollei, Elisabeth Rauh, Martina de Zwaan und Alexandra Martin
12. MglichkeitenzurklinischenDifferenzierung von krperdysmorpher Strung und sozialer Angststrung Anja Grocholewski, Sren Kliem und Nina Heinrichs
13. Die krperdysmorphe Strung Symptomatik und evidenzbasierte Behandlung; Ulrike Buhlmann, Alexandra Martin, Brunna Tuschen-Caffier und Nina Heinrichs
14. SHAME Entwicklung eines Fragebogens zur Erfassung positiver und negativer Aspekte von Scham; Corinna N. Scheel, Caroline Bender, Brunna Tuschen-Caffier und Gitta A. Jacob
15. Therapeutischer Misserfolg in der ambulanten Psychotherapie Daten aus einer verhaltenstherapeutischen Hochschulambulanz; Nicole E. Nelson und Wolfgang Hiller
16. Direkte Vernderungsmessung in der Psychotherapie Der Bochumer Vernderungsbogen-2000 (BVB-2000); Ulrike Willutzki, Dominik lsmann, Dietmar Schulte und Andreas Veith

Journal of Obsessive-Compulsive and Related Disorders

1. N-acetylcysteine augmentation in treatment resistant obsessive compulsive disorder: A case series; Michael Van Ameringen, Beth Patterson, William Simpson, Jasmine Turna

2. Deep brain stimulation for obsessive compulsive disorder: A literature review; M. Mangas, R. Moreira

3. Delivering exposure and ritual prevention for obsessivecompulsive disorder via videoconference: Clinical considerations and recommendations; Elizabeth M. Goetter, James D. Herbert, Evan M. Forman, Erica K. Yuen, Marina Gershkovich, Lisa H. Glassman, Stephanie J. Rabin, Stephanie P. Goldstein

4. Symptom dimensions in OCD and their association with clinical characteristics and comorbid disorders; Lokesh Prabhu, Anish V. Cherian, Biju Viswanath, Thennarasu Kandavel, Suresh Bada Math, Y.C. Janardhan Reddy

5. A comparison of executive function in Body Dysmorphic Disorder (BDD) and Obsessive-Compulsive Disorder (OCD); I. Labuschagne, S.L. Rossell, J. Dunai, D.J. Castle, M. Kyrios

6. Characteristics of animal owners among individuals with object hoarding; Kristin E. Slyne, David F. Tolin, Gail Steketee, Randy O. Frost

7. Development of a scrupulosity severity scale using the Pennsylvania Inventory of Scrupulosity-Revised; Leslie J. Shapiro, Jason W. Krompinger, Christina M. Gironda, Jason A. Elias

8. Preliminary assessment of obsessivecompulsive spectrum disorder scales for DSM-5; Richard T. LeBeau, Emily R. Mischel, Helen B. Simpson, David Mataix-Cols, Katherine A. Phillips, Daniel J. Stein, Michelle G. Craske

9. Using functional analysis to disentangle diagnostic complexities: A case of mucus-related health anxiety; Emily L. Hiatt, Melinda A. Stanley, Ellen J. Teng

10. Effects of tic-related conversation on rate of tics in two siblings; Brad A. Dufrene, T. Steuart Watson, David J. Echevarria, Adam D. Weaver

11. The security motivation system according to Woody and Szechtman and its application to OCD: A critique and alternative; Peter Prudon

12. Predictive validity of explicit and implicit threat overestimation in contamination fear; Jennifer S. Green, Bethany A. Teachman

13. Longer-term effects of inducing harm related intrusions: Implications for research on obsessional phenomena; Noah C. Berman, Amanda W. Calkins, Jonathan S. Abramowitz

14. The Structured Interview for Hoarding Disorder (SIHD): Development, usage and further validation; A.E. Nordsletten, L. Fernndez de la Cruz, A. Pertusa, A. Reichenberg, S.L. Hatch, D. Mataix-Cols

15. Obsessional beliefs, religious beliefs, and scrupulosity among fundamental Protestant Christians; Theodore F. Witzig Jr., C. Alec Pollard

16. Phenomenology and thematic content of intrusive imagery in bowel and bladder obsession; Rosanna Pajak, Christine Langhoff, Sue Watson, Sunjeev K. Kamboj

17. The cost of illness associated with stepped care for obsessive-compulsive disorder; Gretchen J. Diefenbach, David F. Tolin

18. Treating hoarding disorder in childhood: A case study Journal of Obsessive-Compulsive and Related Disorders, Volume 2, Issue 1, January 2013, Pages 62-69 Kaitlin P. Gallo, Lianna A.S. Wilson, Jonathan S. Comer

19. An update on the efficacy of psychological treatments for obsessivecompulsive disorder in adults; Kathryn Ponniah, Iliana Magiati, Steven D. Hollon

20. Development and preliminary psychometric evaluation of a self-rated version of the Family Accommodation Scale for Obsessive-Compulsive Disorder; Anthony Pinto, Barbara Van Noppen, Lisa Calvocoressi

21. Remote treatment of obsessive-compulsive disorder: A randomized controlled trial; Bethany M. Wootton, Blake F. Dear, Luke Johnston, Matthew D. Terides, Nickolai Titov

22. The Spanish version of the Obsessive-Compulsive Inventory-Revised (OCI-R): Reliability, validity, diagnostic accuracy, and sensitivity to treatment effects in clinical samples; Amparo Belloch, Mara Roncero, Gemma Garca-Soriano, Carmen Carri, Elena Cabedo, Hctor Fernndez-lvarez

23. Prevalence, phenomenology and diagnostic criteria of hair-pulling in an Italian non-clinical sample: A preliminary study Original Research Article; Marta Ghisi, Gioia Bottesi, Claudio Sica, Allison J. Ouimet, Ezio Sanavio

24. Risk judgment in ObsessiveCompulsive Disorder: Testing a dual-systems account Gideon Goldin, Mascha van t Wout, Steven A. Sloman, David W. Evans, Benjamin D. Greenberg, Steven A. Rasmussen

25. Changes in protective behavioral strategies and alcohol use among college students; Matthew P. Martensa, Jessica L. Martinb, Andrew K. Littlefieldc, James G. Murphyd, M. Dolores Cimini

26. Stigma and disclosure of intrusive thoughts about sexual themes Angela J. Cathey b,1, Chad T. Wetterneck

27. Risk judgment in ObsessiveCompulsive Disorder: Testing a dual-systems account Gideon Goldin, Mascha van t Wout, Steven A. Sloman , David W. Evans, Benjamin D. Greenberg, Steven A. Rasmussen

28. Do mothers enhance responsibility in children with obsessivecompulsive disorder? A preliminary study of motherchild interactions during a problem solving discussion; Lara J. Farrell, Donna Hourigan, Allison M. Waters

29. Dont judge a book by its cover: ADHD-like symptoms in obsessive compulsive disorder; Amitai Abramovitch, Reuven Dar, Andrew Mittelman, Avraham Schweiger

30. Perceived and actual information processing deficits in nonclinical hoarding; Kristin E. Fitch, Jesse R. Cougle

31. Prevalence and clinical correlates of treatment concerns in a sample of treatment seeking youth with obsessivecompulsive disorder; Robert R. Selles, Karen Rowa, Randi McCabe, Christine Purdon, Eric A. Storch

32. Further evidence for the efficacy of association splitting in obsessive-compulsive disorder. An internet study in a Russian-speaking sample; Steffen Moritz, Rada Russu

33. Neural correlates of symptom reduction after CBT in obsessive-compulsive washersAn fMRI symptom provocation study; Ali Baioui, Juliane Pilgramm, Sabine Kagerer, Bertram Walter, Dieter Vaitl, Rudolf Stark

34. Pediatric obsessivecompulsive disorder: Symptom patterns and confirmatory factor analysis; Gail A. Bernstein, Andrea M. Victor, Peter M. Nelson, Susanne S. Lee
35. Pharmacological and psychological treatments of pathological skin-picking: A preliminary meta-analysis; Bethany L. Gelinas, Michelle M. Gagnon
36. Activities of daily living scale in hoarding disorder; Randy O. Frost, Veselina Hristova, Gail Steketee, David F. Tolin
37. Imagery in mental contamination: A questionnaire study; Anna E. Coughtrey, Roz Shafran, S.J. Rachmany
38. Cognitive functioning in medication-free obsessive-compulsive patients treated with cognitive-behavioural therapy Original Research Article Journal of Obsessive-Compulsive and Related Disorders, Volume 2, Issue 3, July 2013, Pages 241-248 Ulrich Voderholzer, Caroline Schwartz, Tobias Freyer, Bartosz Zurowski, Nicola Thiel, Nirmal Herbst, Karina Wahl, Andreas Kordon, Fritz Hohagen, Anne Katrin Kuelz

International Journal of Psychology and Psychological Therapy

1. An Empirical Investigation of Hierarchical versus Distinction Relations in a Self-based ACT Exercise Mairad Foody, Yvonne Barnes-Holmes, Dermot Barnes-Holmes
2. The Effectiveness of Logotherapy Program in Alleviating the Sense of Meaninglessness of Paralyzed In-patients Angelina M. Julom, Rosalito de Guzmn
3. Value Change and Post-modernism: A Preliminary Study of a German Sample Yolanda Alonso, F Rosa Jimnez-Lpez, Gustavo Garca-Vargas, Jess Gil Roales-Nieto
4. Revisiting Kazdin (1980): Contemporary Treatment Acceptability for Problem Behavior in Children James W. Diller, Robert M. Brown, Connor H. G. Patros
5. Facial Feedback in Implicit Sequence Learning Christina Bermeitinger, Anna-Maria Machmer, Julia Schramm, Dennis Mertens D. Luisa Wilborn, Larissa Bonin, Heidi Femppel, Friederike Koch
6. Early maladaptive schemas and interpersonal problems: A circumplex analysis of the YSQ-SF Jens C. Thimm
7. A New Approach to Explain the Link between Social Support and Depression in a 2-years Arthritis Rheumatoid Sample. Is there any Moderation Effect of Acceptance? Joana Costa, Jos Pinto Gouveia
8. Parents beliefs on the causes of child maltreatment Maria Manuela Calheiros

2 Rejection of linear regression on basis of correct assumptions

Zeitschrift fur Klinische Psychologie und Psychotherapie

1. Symptomkategorien der Zwangsstrungspezifischodergeneralisiert? Ein Vergleich zwischen der Yale-Brown Obsessive-Compulsive Skala (Y-BOCS) und dem Vancouver Obsessional Compulsive Inventar (VOCI); Gudrun Sartory, Gisela Rper, Reinhard Pietrowsky, Jan Cwik1 und Michael Zaudig

Journal of Obsessive-Compulsive and Related Disorders

1. Family environment in adolescent trichotillomania Nancy J. Keuthena, Jeanne Famaa, Erin M. Altenburgera, Amanda Allenb, Anna Raff, David Paulsa

3 Rejection of linear regression on basis of not meeting incorrect assumptions

Zeitschrift fur Klinische Psychologie und Psychotherapie

1. Erfassung aggressionsauslsender und -aufrechterhaltender Faktoren bei Kindern mit oppositionellen und aggressiven Verhaltensstrungen im Eltern-, Lehrer- und Selbsturteil Zur Reliabilitt und Validitt des Fragebogens zum aggressiven Verhalten von Kindern (FAVK); ChristinaBenesch, Anja Grtz-Dorten, DieterBreuer und Manfred Dpfner

4 Correct linear regression

5 Mentioned all correct assumptions but not if the normality assumption was tested on the residuals or on X or Y

6 Did not test all but some correct assumptions, included neither normality of variables nor residuals

7 Use of linear regression but no indication if any or which assumptions were tested

Zeitschrift fur Klinische Psychologie und Psychotherapie

1. Belastungen in der Kindheit und dissoziative Symptomatik bei Patienten mit psychischen Strungen; Johanna Fiess, Astrid Steffen, Christian Pietrek und Brigitte Rockstroh
2. Die Rolle sozialer Untersttzung, Religiositt und Achtsamkeit im Rahmen der Krankheitsverarbeitung bei Brustkrebspatientinnen; Marion Hofner und Johannes Michalak
3. Die Interpersonale Theorie suizidalen Verhaltens Eine systematische bersichtsarbeit Sarah Wachtel und Tobias Teismann
4. Es ging mir schlechter, als ich dachte! Retrospektive Symptomeinschtzung und Psychotherapieerfolg Dominik lsmann und Thomas Fydrich
5. Die gemeinsamen und spezifischen Komponenten von Depression und Angst berprfung eines Modells zur Faktorenstruktur depressiver und ngstlicher Symptome und deren Beziehung zu Affekt; Christian Baumann, Benedikt Klauke, Katharina Domschke, Frauke Fassbinder, Nina Gartmann, Raffael Kalisch, Andreas Reif, Jrgen Deckert und Paul Pauli

Journal of Obsessive-Compulsive and Related Disorders

1. Fear of self and obsessionality: Development and validation of the Fear of Self Questionnaire; Frederick Aardema, Richard Moulding, Adam S. Radomsky, Guy Doron, Jude Allamby, Eman Souki

2. Gastro-intestinal conditions in patients with obsessive compulsive disorder; Ellemijn A. Kuiper, Neeltje M. Batelaan, Patricia van Oppen, Gert-Jan Hendriks, Anton J.L.M. van Balkom

3. An examination of excessive acquisition in hoarding disorder; Randy O. Frost, Elizabeth Rosenfield, Gail Steketee, David F. Tolin

4. Symmetry concerns as a symptom of body dysmorphic disorder; Ashley S. Hart, Katharine A. Phillips

5. The obsessive-compulsive trait of Incompleteness in parents of children with autism spectrum disorders; Patricia H. Kloosterman, Laura J. Summerfeldt, James D.A. Parker, Jeanette J.A. Holden

6. Impulsivity in hoarding; Jessica L. Rasmussen, Timothy A. Brown, Gail S. Steketee, David H. Barlow

7. Lutheran clergy members' responses to scrupulosity: The effects of moral thoughtaction fusion and liberal vs. conservative denomination; Brett J. Deacon, Amanda M. Vincent, Annie R. Zhang

8. Do people with hoarding disorder under-report their symptoms? Jennifer DiMauro, David F. Tolin, Randy O. Frost, Gail Steketee

9. The Icelandic version of the dimensional obsessive compulsive scale (DOCS) and its relationship with obsessive beliefs; Ragnar P. lafsson, Jhann B. Arngrmsson, Pll rnason, rinn Kolbeinsson, Paul M.G. Emmelkamp, rni Kristjnsson, Danel . lason

10. Disgust propensity and sensitivity: Differential relationships with obsessive-compulsive symptoms and behavioral approach task performance; Amy R. Goetz, Han-Joo Lee, Jesse R. Cougle, Jennifer E. Turkel

11. Thought control moderates the relation between autogenous intrusions and the severity of obsessional symptoms: Further support for the autogenousreactive model of obsessions; Thomas A. Fergus

12. A multitrait-multimethod matrix investigation of hoarding Original Research Article Journal of Obsessive-Compulsive and Related Disorders, Volume 2, Issue 3, July 2013, Pages 273-280 Joseph F. Meyer, Randy O. Frost, Timothy A. Brown, Gail Steketee, David F. Tolin

13. Childhood trauma and thought action fusion: A multi-method examination Journal of Obsessive-Compulsive and Related Disorders, Volume 2, Issue 1, January 2013, Pages 43-47 Noah C. Berman, Michael G. Wheaton, Jonathan S. Abramowitz

14. Mediation and interaction effects of doubt, dysfunctional beliefs and memory confidence on the compulsion to check; Carrie Cuttler, Gillian M. Alcolado, Steven Taylor

15. Autogenous/reactive obsessions and their relationship with negative self-inferences; Jang-Won Seo, Seok-Man Kwon

16. Psychometric properties of the Obsessive-Compulsive Inventory Revised in African Americans with and without obsessive-compulsive disorder; Monnica Williams, Darlene M. Davis, Michel A. Thibodeau, Nicholas Bach

International Journal of Psychology and Psychological Therapy

1. Drive for thinness as a womens strategy to avoid inferiority Cludia Ferreira* Jos Pinto Gouveia Cristiana Duarte
2. Psychometric Evaluation of a Shortened Version of the 40-item Defense Style Questionnaire; Carine Saint-Martin , Marjorie Valls , Amelie Rousseau , Stacey Callahan , Henri Chabrol

8 Assumed/tested normally distributed X but not the normality of the residuals

Journal of Obsessive-Compulsive and Related Disorders

1. Fathers' not just right experiences predict obsessivecompulsive symptoms in their sons: Family study of a non-clinical Italian sample; Claudio Sica, Corrado Caudek, Gioia Bottesi, Eleonora De Fazio, Marta Ghisi, Igor Marchetti, Antonella Orsucci
2. Environmental factors as potential determinants of premonitory urge severity in youth with Tourette syndrome; Matthew R. Capriotti, Flint M. Espil, Christine A. Conelea, Douglas W. Woods
3. Responsibility, metacognition and unrealistic pessimism in obsessivecompulsive disorder; Helen Niemeyer, Steffen Moritz, Reinhard Pietrowsky
4. Clinical correlates of functional impairment in children and adolescents with obsessive-compulsive disorder; Joshua M. Nadeau n, Adam B. Lewin, Elysse B. Arnold, Erika A. Crawford, Tanya K. Murphy, Eric A. Storch

9 Assumed/tested normally distributed Y but not the normality of the residuals

Journal of Obsessive-Compulsive and Related Disorders

1. Gender specific short stature in male adolescents with obsessive-compulsive disorder; Yu-Pei Hu, Rebecca Wehrly, Tristan Gorrindo, Dianne M. Hezel, Christina Gironda, Michael A. Jenike, S. Evelyn Stewart

10 Assumed/tested normally distributed X and Y but not the normality of the residuals

11 Assumed/tested normally distributed variables but did not indicate if X or Y or both and did not test the normality of the residuals

12 Other misconceptions about assumptions

Part IV
Q4 top 3: Internet Journal of Mental Health, Indian Journal of Psychological Medicine, Behaviour Change

1 No Model of Interest

Indian Journal of Psychological Medicine

1. Olanzapine-induced tardive oculogyric crises; Amar D Bavle, Girish M. N. Kumar
2. Is semen loss syndrome a psychological or physical illness? A case for conflict of interest; Shivanand Kattimani, Vikas Menon, Manohar Kant Shrivastava
3. Gratification disorder mimicking childhood epilepsy in an 18-month-old Nigerian girl: A case report and review of the literature; Aliyu Ibrahim, Belonwu Raymond
4. Linezolid-induced near-fatal serotonin syndrome during escitalopram therapy: Case report and review of literature; Ranganath R Kulkarni, Pratibha R Kulkarni
5. Transient eating problems in an adolescent without body image disturbances: A diagnostic quandary; Siddharth Sarkar, Susanta Kumar Padhy, Pradyumna Rao, Sunil Gupta
6. Early onset obsessive compulsive disorder with obsessive slowness: A case report and demonstration of management; Ashish Kumar Mittal, Pradipta Majumder, Alok Agrawal, Mamta Sood, Sudhir Kumar Khandelwal
7. Cannabinoid hyper-emesis syndrome: An enigma; Neeraj Gupta, Oladele Ojo, Kanishka Muruthettuwegama
8. Atypical charles bonnet syndrome; Priti Arun, Rajan Jain, Vaibhav Tripathi
9. Recurrent dissociative fugue; Abhishek Mamarde, Praveen Navkhare, Amrita Singam, Akash Kanoje
10. Tiapride for the treatment of auditory hallucinations in schizophrenia; Sagar Karia, Nilesh Shah, Avinash De Sousa, Sushma Sonavane

11. Adolescent male peer sexual abuse: An issue often neglected; Girish H Banwari
12. Coping styles and its association with sources of stress in undergraduate medical students; Sandhya Cherkil, Seby J Gardens, Deepak Kuttikatt Soman
13. Factorial validity and reliability of the tamil version of multidimensional scale of perceived social support among a group of participants in University Malaya Medical Centre, Malaysia; Ng Chong Guan, Abdul Rasyid Sulaiman, Loh Huai Seng, Anne Yee Hway Ann, Suzaily Wahab, Subash Kumar Pillai
14. Health status of the prisoners in a central jail of south India; Sunil D Kumar, Santosh A Kumar, Jayashree V Pattankar, Shrinivas B Reddy, Murali Dhar
15. Effect of educational module on knowledge of primary school teachers regarding early symptoms of childhood psychiatric disorders; Liza Thankam Daniel, Sandhya Gupta, Rajesh Sagar
16. Effect of educational module on knowledge of primary school teachers regarding early symptoms of childhood psychiatric disorders; Liza Thankam Daniel, Sandhya Gupta, Rajesh Sagar
17. Schedule of factors influencing access to psychiatric treatment in persons with schizophrenia: Validity and pilot testin; K Shanivaram Reddy, Jagadisha Thirthalli, C Naveen Kumar, N Krishna Reddy, NR Renukadevi, Vikram Singh Rawat, Jayashree Ramkrishna, Bangalore N Gangadhar
18. Validation of Hindi translation of SRPB facets of WHOQOL-SRPB scale; Sandeep Grover, Ruchita Shah, Parmanand Kulhara
19. Caregivers burden of patients with schizophrenia and bipolar disorder: A sectional study; Vasudeva, Chandra K Sekhar, Prasad G Rao
20. Impact of comorbidity on three month follow-up outcome of children with ADHD in a child guidance clinic: Preliminary report; Rangan Srinivasaraghavan, Subramanian Mahadevan, Shivanand Kattimani
21. Assessment of cognitive behavioral therapy on quality of life of patients with chronic war-related post-traumatic stress disorder; Mohammadjavad Ahmadizadeh, Khodabakhsh Ahmadi, Jafar Anisi, Amir Bahrami Ahmadi
22. Mental health care of older people: can the district mental health program of India make a difference? Harish M Tharayil, Anish Thomas, Bindu V Balan, KS Shaji
23. Dhat syndrome: A review of the world literature; Koushik Sinha Deb, Yatan Pal Singh Balhara
24. Meige's syndrome: Rare neurological disorder presenting as conversion disorder; Mohapatra Debadatta, Ajay K Mishra
25. Cotard syndrome with catatonia: Unique combination; Aniruddha Basu, Priti Singh, Rajiv Gupta, Sandeep Soni
26. Bupropion-warfarin combination: A serious complication; Amar D Bavle, Akshay S Phatak

27. A classical case of bulimia nervosa from India; Piyali Mandal, S Arumuganathan, Rajesh Sagar, Pakhi Srivastava

28. Therapist concerns and process issues in grappling with functional autobiographical amnesia; Ravikesh Tripathi, Srikala Bharath, Geetha Desai, Seema Mehrotra

29. Capacity building in mental health research: The way forward; KS Shaji

30. Objective structured clinical examination and its impact on clinical and interpersonal skills: Follow-up study; Manoj Kumar Sharma, Prabha S Chandra, SK Chaturvedi

31. Role of hypothalamic-pituitary-adrenal-axis in affective disorders: Anti-depressant and anxiolytic activity of partial 5-HT1A agonist in adrenalectomised rats; Deepali Gupta, Mahesh Radhakrishnan, Shvetank Bhatt, Yeshwant Kurhe

32. Karnataka state telemedicine project: Utilization pattern, current, and future challenges; Bharath Holla, Biju Viswanath, Shanthaveeranna Neelaveni, T Harish, Channaveerachari Naveen Kumar, Suresh Bada Math

33. Bizarre delusions: A qualitative study on Indian schizophrenia patients; Sreeja De, Triptish Bhatia, Pramod Thomas, Satabdi Chakraborty, Shiv Prasad, Rajesh Nagpal, Vishwajit L Nimgaonkar, Smita N Deshpande

34. Parent-of-origin effect in schizophrenia and non-affective psychoses: Evidence from dermatoglyphics; Anjith Divakaran, Janardhanan C Narayanaswamy, Sunil V Kalmadi, Vidya Narayan, Naren P Rao, Ganesan Venkatasubramanian

35. Parent-of-origin effect in schizophrenia and non-affective psychoses: Evidence from dermatoglyphics; Anjith Divakaran, Janardhanan C Narayanaswamy, Sunil V Kalmadi, Vidya Narayan, Naren P Rao, Ganesan Venkatasubramanian

36. Tramadol use in premature ejaculation: Daily versus sporadic treatment; Amil H Khan, Deepa Rasaily

37. Family burden in patient with schizophrenia and depressive disorder: A comparative study; Sateesh R Koujalgi, Shobhadevi R Patil

38. Lomotil (diphenoxylate) dependence in India; Aseem Mehra, Siddharth Sarkar, Debasish Basu

39. Metabolic syndrome in schizophrenia; Nidhi Malhotra, Sandeep Grover, Subho Chakrabarti, Parmanand Kulhara

40. Disulfiram induced reversible hypertension: A prospective case study and brief review; Ranganath R Kulkarni, Bhavya K Bairy

41. Delusional procreation syndrome: Report from TURUVECARE community intervention program; Narayana Manjunatha, Shanivaram K Reddy, NR Renuka Devi, Vikram Rawat, Somashekar Bijjal, Naveen C Kumar, KV Kishrore Kumar, Jagadisha Thirthalli, BN Gangadhar

42. Zolpidem-induced hallucinations: A brief case report from the indian subcontinent; Gurvinder Pal Singh, Neeraj Loona

43. Wires in the body: A case of factitious disorder; Koushik Sinha-Deb, Siddharth Sarkar, Mamta Sood, Sudhir K Khandelwal
44. Electro convulsive therapy in psychiatric manifestations in Wilson's disease; Parth Vaishnav, HA Gandhi
45. Sexual abuse in 8-year-old child: where do we stand legally? Prakash Balkrishna Behere, Akshata Nandu Mulmule
46. Relationship of personality dimensions and aggression in romantic relationship among youth; Manoj K Sharma, Mohan Raju
47. A computerized stroop test for the evaluation of psychotropic drugs in healthy participants; Raveendranadh Pilli, MUR Naidu, Usha Rani Pingali, JC Shobha, A Praveen Reddy
48. A descriptive study of clinical, hematological, and biochemical parameters of inhalant users seeking treatment at a tertiary care center in India; Rizwana Quraishi, Raman Deep Pattanayak, Raka Jain, Anju Dhawan
49. Metabolic syndrome in drug-nave patients with depressive disorders; Sandeep Grover, Naresh Nebhinani, Subho Chakrabarti, Ajit Avasth, Parmanand Kulhara
50. A comparative study of attitudes toward psychiatry among nursing students across successive training years; Yatan Pal Singh Balhara, Shachi Mathur
51. Perception and attitude towards mental illness in an urban community in South Delhi - A community based study; Harshal Salve, Kiran Goswami, Rajesh Sagar, Baridalyne Nongkynrih, Vishnubhatla Sreenivas
52. Growing up with a parent having schizophrenia: Experiences and resilience in the offsprings, Hesi S Herbert, M Manjula, Mariamma Philip
53. Effects of psychopathology, functioning and anti-psychotic medication adherence on caregivers' burden in schizophrenia; VO Lasebikan, OO Ayinde
54. How to calculate sample size for different study designs in medical research? Jaykaran Charan, Tamoghna Biswas
55. Physician-assisted suicide and euthanasia in Indian context: Sooner or later the need to ponder! Farooq Khan, George Tadros
56. An unusual case of suicide attempt using intravenous injection of kerosene

    Sushmitha Jayaprasad, Vijayashankar Metikurke
57. Status epilepticus following electroconvulsive therapy; TM Omprakash, Arindam Chinmoy Chakrabarty, P Surender
58. Cognitive therapy of obsessive compulsive disorder with chronic tic disorder; Sudhir Hebbar
59. Working with art in a case of schizophrenia; Konrad J Noronha
60. Risperidone associated paralytic ileus in schizophrenia Parthasarathy Ramamourthy, Arunkumar Kumaran, Shivanand Kattimani
61. Alternative psychosis - Is it a defined clinical entity? Girish H Banwari, Chirag D Parmar, Dhiraj D Kandre

62. Use of electroconvulsive therapy in the presence of GLAUCOMA: A case report and review of literature; Jitender Aneja, Sandeep Grover, Munish Agarwal, Sushmita Kaushik
63. Comorbidity of psychiatric and personality disorders in first suicide attempters; K Nagaraja Rao, Ranganath R Kulkarni, Shamshad Begum
64. Neurohemodynamic correlates of washing symptoms in obsessive-compulsive disorder: A pilot fMRI study using symptom provocation paradigm; Sri Mahavir Agarwal, Dania Jose, Upasana Baruah, Venkataram Shivakumar, Sunil Vasu Kalmady, Ganesan Venkatasubramanian, David Mataix-Cols, Yemmigannur Chandrashekhar Janardhan Reddy
65. Family burden in caregivers of schizophrenia patients: Prevalence and socio-demographic correlates; Victor Olufolahan Lasebikan, Olatunde Olayinka Ayinde
66. Anxiety disorders in bipolar I mania: Prevalence, effect on illness severity, and treatment implications; Anindya Das
67. A follow-up study of academic functioning and social adjustment in children with attention deficit hyperactivity disorder; Jasmin Garg, Priti Arun
68. Psychosocial stressors and patterns of coping in adolescent suicide attempters; Mathew A, Nanoo S
69. Assessing perceived stress in medical personnel: In search of an appropriate scale for the Bengali population; Amrita Chakraborti, Prasenjit Ray, Debasish Sanyal, Rajarshi Guha Thakurta, Amit K Bhattacharayya, Asim Kumar Mallick, Ranjan Das, Syed Naiyer Ali
70. How to teach psychiatry to medical undergraduates in India?: A model; SM Manohari, R Johnson Pradeep, Ravindra Baburao Galgali
71. Psychosocial issues of children infected with HIV/AIDS; MN Vranda, SN Mothi
72. Long-acting preparations in substance abuse management: A review and update; Aditya Hegde, Shubh Mohan Singh, Siddharth Sarkar
73. Adjustment disorder: Current diagnostic status; Bichitra Nanda Patra, Siddharth Sarkar
74. Metabolic syndrome in alcohol-dependent men: A cross-sectional study; Jitender Aneja, Debasish Basu, Surendra Kumar Mattoo, Krishan Kumar Kohli
75. A study of profile of disability certificate seeking patients with schizophrenia over a 5 year period; Yatan Pal Singh Balhara, Rohit Verma, Smita N Deshpande
76. Thalamic shape abnormalities in antipsychotic nave schizophrenia; Vijay Danivas, Sunil V Kalmady, Ganesan Venkatasubramanian, Bangalore N Gangadhar
77. A descriptive analysis of patients presenting to psychosexual clinic at a tertiary care center; Rohit Verma, Shaily Mina, Shiraz Ul-Hassan, Yatan Pal Singh Balhara
78. A comparative study of simple auditory reaction time in blind (congenitally) and sighted subjects; Pritesh Hariprasad Gandhi, Pradnya A Gokhale, HB Mehta, CJ Shah
79. Prevalence of depression in patients with type ii diabetes mellitus and its impact on quality of life; Ranjan Das, Omprakash Singh, Rajarshi Guha Thakurta1, MR Khandakar, SN Ali, Asim Kumar Mallick, Paromita Roy, Amit K Bhattacharrya

80. Psychiatric morbidity among inmates of leprosy homes; KC Jindal, Gurvinder Pal Singh, Varinder Mohan, BB Mahajan
81. An exploratory analysis of personality factors contributed to suicide attempts; P. N. Suresh Kumar, V Rajmohan, K Sushil
82. A psychological study of stress, personality and coping in police personnel; Ravneet Kaur, Vamsi K Chodagiri, Narasimha K Reddi

The Internet Journal of Mental Health

1. Behavioral Disturbance From Suspected Frontotemporal Dementia And Comorbid Paraphilia: A Case Report; J R Scarff, R Baweja, R Mogallapu, S Burton, S Lippmann

Behaviour Change

1. Interpersonal Difculties as an Underlying Mechanism in the Anxiety-Depression Association Andres G. Viana and Erin N. Stevens
2. Parent-Endorsed Reasons for Not Completing Homework in Group-Based Behavioural Parent Training for High-Risk Families of Youth With ADHD Anil Chacko, Lindsay Anderson, Brian T. Wymbs and Frances A. Wymbs
3. Using a Behavioural Family Intervention to Produce a Three-Generational Benet on Family Outcomes: A Case Report James N. Kirby and Matthew R. Sanders
4. A Preliminary Study of Anxiety, Negative Affect, Experiential Avoidance, and Delaying of Aversive Events Kristalyn Salters-Pedneault and James W. Diller
5. CBT Guided Self-Help Compares Favourably to Gold Standard Therapist-Administered CBT and Shows Unique Benets Over Traditional Treatment Margaret Priemer and France Talbot
6. Towards a Unied Worry Exposure Protocol for Generalised Anxiety Disorder: A Pilot Study Cameron McIntosh and Rocco Crino
7. The Common Fears and Their Origins Among Turkish Children and Adolescents Begm Serim-Yldz, zgr Erdur-Baker and Asl Bugay
8. Further Evidence That Repeated Checking Leads to Reduced Memory Condence, Vividness and Detail: New Evidence That Repeated Object Exposure Also Results in Memory Distrust Meredith Medway and Mairwen K. Jones
9. Assessing Randomised Clinical Trials of Cognitive and Exposure Therapies for Gambling Disorders: A Systematic Review David P. Smith, Kirsten I. Dunn, Peter W. Harvey, Malcolm W. Battersby and Rene G. Pols
10. A Proof of Concept for Using Brief Dialectical Behavior Therapy as a Treatment for Problem Gambling Darren R. Christensen, Nicki A. Dowling, Alun C. Jackson, Meredith Brown, James Russo, Kate L. Francis and Azusa Umemoto
11. Review of the Theoretical, Empirical, and Clinical Status of Adaptive and Maladaptive Perfectionism Alice Lo and Maree J. Abbott
12. Contingency Management to Induce Exercise Among College Students Jessica G. Irons, Derek A. Pope, Allyson E. Pierce, Ryan A. Van Patten and Brantley P. Jarvis

13. Impact of Cognitive Behaviour Therapy Via Mail for Cessation of Benzodiazepine Use: A Series of Case Reports Jannette M. Parr, David J. Kavanagh, Ross McD. Young, Barbara Stubbs and Nick Bradizza
14. Uncontrolled, Repetitive Eating of Small Amounts of Food or Grazing: Development and Evaluation of a New Measure of Atypical Eating Brigitte Lane and Marianna Szab
15. Using Escape Extinction and Reinforcement to Increase Eating in a Young Child with Autism Lilly T. D. Bui, Dennis W. Moore and Angelika Anderson
16. Cognitive Behaviour Therapy for Problem Gamblers: A Clinical Outcomes Evaluation Barry Tolchard and Malcolm W. Battersby
17. Unresolved Issues Regarding Collaborative Empiricism in Cognitive and Behavioural Therapies: An Expert Panel Discussion at AACBT Nikolaos Kazantzis, Arthur Freeman, Alan E. Fruzzetti, Jacqueline B. Persons and Mervin Smucke
18. Interpersonal and Intrapersonal Functions of Deliberate Self-Harm (DSH): A Psychometric Examination of the Inventory of Statements About SelfInjury (ISAS) Scale Rebecca Kortge, Tanya Meade and Alan Tennant

2 Rejection of linear regression on basis of correct assumptions

3 Rejection of linear regression on basis of not meeting incorrect assumptions

4 Correct linear regression

5 Mentioned all correct assumptions but not if the normality assumption was tested on the residuals or on X or Y

6 Did not test all but some correct assumptions, included neither normality of variables nor residuals

7 Use of linear regression but no indication if any or which assumptions were tested

The Internet Journal of Mental Health

1. Development And Validation Of The Japanese Coping Scale Administered Over The Internet; K Nakano

Behaviour Change

1. Autobiographical Memory in Children and Adolescents With Acute Stress and Chronic Posttraumatic Stress Disorder Reginald D.V. Nixon, Shelly-Anne Ball, Jisca Sterk, Talitha Best and Lisa Beatty

2. Are Positive Beliefs about Post-Event Processing Related to Social Anxiety? Brian Fisak, Jr. and Amanda N. Hammond

8 Assumed/tested normally distributed X but not the normality of the residuals

9 Assumed/tested normally distributed Y but not the normality of the residuals

10 Assumed/tested normally distributed X and Y but not the normality of the residuals

11 Assumed/tested normally distributed variables but did not indicate if X or Y or both and did not test the normality of the residuals

12 Other misconceptions about assumptions
