# Supplementary material for: Regression assumptions in clinical psychology research practice—a systematic review of common misconceptions
Source: PeerJ. 2017 May 16;5:e3323. doi: 10.7717/peerj.3323 (PMC5436580; doi:10.7717/peerj.3323)
Supplement: Supplemental Information 3 [file peerj-05-3323-s003.pdf]

List of websites of all journals

Q1.1 Annual Review of Clinical Psychology
http://www.annualreviews.org/toc/clinpsy/9/1
Q1.2 Clinical Psychology Review
https://www.journals.elsevier.com/clinical-psychology-review/
Q1.3 Journal of Consulting and Clinical Psychology
Retrieved from PsycARTICLES:
http://www.apa.org/pubs/databases/psycarticles/coverage-list.aspx

Q2.1 International Psychogeriatrics
https://www.cambridge.org/core/journals/international-psychogeriatrics
Q2.2 Journal of Attention Disorders
http://journals.sagepub.com/loi/jada?expanded=2013
Q2.3 The American Journal of Drug and Alcohol Abuse
http://www.tandfonline.com/loi/iada20

Q3.1 Zeitschrift für Klinische Psychologie und Psychotherapie
http://econtent.hogrefe.com/loi/zkp?expanded=2013
Q3.2 Journal of Obsessive-Compulsive and Related Disorders
https://www.journals.elsevier.com/journal-of-obsessive-compulsive-and-related-disorders/
Q3.3 International Journal of Psychology and Psychological Therapy
http://www.ijpsy.com/

Q4.1 The Internet Journal of Mental Health
http://ispub.com/IJMH
Q4.2 Indian Journal of Psychological Medicine
http://www.ijpm.info/
Q4.3 Behaviour Change
https://www.cambridge.org/core/journals/behaviour-change/all-issues
